# Development of a rational framework for the therapeutic efficacy of fecal microbiota transplantation for calf diarrhea treatment

**DOI:** 10.1186/s40168-021-01217-4

**Published:** 2022-02-21

**Authors:** Jahidul Islam, Masae Tanimizu, Yu Shimizu, Yoshiaki Goto, Natsuki Ohtani, Kentaro Sugiyama, Eriko Tatezaki, Masumi Sato, Eiji Makino, Toru Shimada, Chise Ueda, Ayumi Matsuo, Yoshihisa Suyama, Yoshifumi Sakai, Mutsumi Furukawa, Katsuki Usami, Hiroshi Yoneyama, Hisashi Aso, Hidekazu Tanaka, Tomonori Nochi

**Affiliations:** 1 International Education and Research Center for Food and Agricultural Immunology, Graduate School of Agricultural Science, Tohoku University, 468-1 Aoba, Aramaki, Aoba-ku, Sendai, Miyagi 980-8572 Japan; 2 East Veterinary Clinical Center, Chiba Prefectural Federation of Agricultural Mutual Aid Association, 1533 Naruto, Sanmu Chiba, 289-1326 Japan; 3 North Veterinary Clinical Center, Chiba Prefectural Federation of Agricultural Mutual Aid Association, 99-1 Nira, Katori, Chiba, 289-0407 Japan; 4 West Veterinary Clinical Center, Chiba Prefectural Federation of Agricultural Mutual Aid Association, 154-11, Shisui-machi, Imba-gun, Chiba 285-0902 Japan; 5 Central Veterinary Clinical Center, Chiba Prefectural Federation of Agricultural Mutual Aid Association, 736 Amoda, Ichihara, Chiba 299-0126 Japan; 6 Division of Mucosal Vaccines, International Research and Development Center for Mucosal Vaccines, The Institute of Medical Science, The University of Tokyo, 4-6-1 Shirokanedai, Minato-ku, Tokyo 108-8639 Japan

**Keywords:** Calf diarrhea, FMT, Microbiome, Metabolites

## Abstract

**Background:**

Establishing fecal microbiota transplantation (FMT) to prevent multifactorial diarrhea in calves is challenging because of the differences in farm management practices, the lack of optimal donors, and recipient selection. In this study, the underlying factors of successful and unsuccessful FMT treatment cases are elucidated, and the potential markers for predicting successful FMT are identified using fecal metagenomics via 16S rRNA gene sequencing, fecal metabolomics via capillary electrophoresis time-of-flight mass spectrometry, and machine learning approaches.

**Results:**

Specifically, 20 FMT treatment cases, in which feces from healthy donors were intrarectally transferred into recipient diarrheal calves, were conducted with a success rate of 70%. *Selenomonas* was identified as a microorganism genus that showed significant donor–recipient compatibility in successful FMT treatments. A strong positive correlation between the microbiome and metabolome data, which is a prerequisite factor for FMT success, was confirmed by Procrustes analysis in successful FMT (*r* = 0.7439, *P* = 0.0001). Additionally, weighted gene correlation network analysis confirmed the positively or negatively correlated pairs of bacterial taxa (family *Veillonellaceae*) and metabolomic features (i.e., amino acids and short-chain fatty acids) responsible for FMT success. Further analysis aimed at establishing criteria for donor selection identified the genus *Sporobacter* as a potential biomarker in successful donor selection. Low levels of metabolites, such as glycerol 3-phosphate, dihydroxyacetone phosphate, and isoamylamine, in the donor or recipients prior to FMT, are predicted to facilitate FMT.

**Conclusions:**

Overall, we provide the first substantial evidence of the factors related to FMT success or failure; these findings could improve the design of future microbial therapeutics for treating diarrhea in calves.

Video abstract

**Supplementary Information:**

The online version contains supplementary material available at 10.1186/s40168-021-01217-4.

## Background

Fecal microbiota transplantation (FMT), in which fecal contents from healthy donors are transplanted into diseased patients with the intention of normalizing or restoring healthy gut microbiota, is considered a promising therapeutic for dysbiosis-related diseases [1, 2]. Infections caused by recurrent *Clostridioides difficile* can be treated using FMT with a high success rate [3, 4], and FMT is used as a treatment option on patients with IBD, IBS, and autoimmune disorders [5]. Recently, the efficacy of FMT for the treatment of multifactorial calf diarrhea (CD) has also been confirmed [6]. However, maximizing the effects of FMT as a treatment for CD remains a challenge because the donor or recipient may be selected inappropriately due to gut microbiota compositions varying even within healthy populations as a result of environmental factors, farm management, and calf age [3]. These obstacles may increase the lack of reproducibility or the risk of FMT failure when attempting to prevent CD.

CD is a common enteric disease that causes enormous financial losses in the livestock industry worldwide due to high morbidity and mortality [7]. Infectious CD is mainly caused by infection from viruses (e.g., rotavirus and coronavirus), bacteria (e.g., enterotoxigenic *Escherichia coli*, *Salmonella*, and *Clostridium perfringens*), protozoa (e.g., *Cryptosporidium parvum* and coccidia), or a combination of such pathogens [8, 9]. However, these enteropathogens are also found in many healthy calves, indicating that their presence is not always responsible for the occurrence of diarrhea [10]. Therefore, clinical veterinarians face a huge challenge to provide correct CD diagnoses; indeed, misdiagnosis may promote the improper use of antibiotics and emergence of antibiotic-resistant bacteria [11, 12].

Microbial symbiosis in the gastrointestinal tract is vital for host regulation of mucosal immunity and nutrients and for providing resistance against pathogen colonization [13]. Microbial colonization of newborns is pivotal to healthy development. Following FMT, the beneficial microbiota of healthy donors can restore the microbial community of recipient calves suffering from CD. To ensure the efficacy and durability of FMT as a disease cure, both the microbial consortium from the donor and existing endogenous microbiota in the recipient must function collaboratively [14]. Therefore, although FMT may be an alternative therapy for CD prevention, the following must still be investigated and understood: (1) the beneficial microorganisms present in the feces of FMT donors, (2) the functional microorganisms and metabolites that lead to success (or failure) of FMT, and (3) the characteristics of FMT recipients that facilitate the engraftment and maintenance of donor-derived beneficial microorganisms and metabolites.

To establish FMT as a common therapeutic with high repeatability and reproducibility in veterinary medicine, the logistics of selecting donors and optimizing efficacy must be developed further. Typical approaches to select donors for experimental FMT treatments involve using a single donor or randomly selecting multiple donors from a set of screened potential individuals [15], which is often impractical. Therefore, we aimed to address this issue by introducing a random forest (RF) classification method. The RF classification is an effective machine learning approach that can be used to elucidate the relationship between high-dimensional microbiota data and disease attributes. With this model, structural changes in existing microbiota data are analyzed to make new predictions and evaluate the subset of microbial taxa with relative abundances that are positively or negatively correlated with disease- or therapeutic-related target variables [16, 17]. Therefore, in the present study, microbial composition data from FMT treatments along with additional field data obtained from a sufficient with or without diarrhea were subjected to RF classification to determine the optimal donor prediction for successful FMT when it is applied in future cases.

## Methods

### Animals

To elucidate the efficacy of FMT in intractable diarrhea treatments, healthy calves (*n* = 20) and calves suffering from intractable diarrhea (*n* = 20) were randomly selected as donors and recipients, respectively, in Chiba, Japan. We performed 2, 3, 2, 4, 3, and 6 trials in 6 individual farms. All donors and recipient pairs were experimentally selected from the same farm to avoid unexpected farm-to-farm bacterial/viral transmission. The absence of enteric pathogens in the feces of donors was confirmed at the Sanritsu Zelkova Veterinary Laboratory before the FMT treatments were administered. Specifically, fecal samples collected from FMT donors and recipients were subjected to etiologic evaluation against 10 major enteropathogens that often cause CD (i.e., bovine leukemia virus, bovine viral diarrhea, rotavirus, *C. perfringens*, *C. parvum*, coccidia, *Salmonella* spp., coronavirus, pathogenic *E. coli*, and nematodes). The severity of diarrhea in recipients was graded according to fecal consistency. Fecal scores were assigned as follows: 1 = normal (retains form), 2 = soft (flows across a surface), 3 = muddy (liquid), and 4 = severe diarrhea (very watery). To estimate the diarrheal condition as well as colonic motility, the water content in feces was investigated by comparing the weight before and after freeze-drying and using the following calculation: water content (%) = 100 × (wet weight − dry weight)/wet weight according to a previous study [18]. FMT-recipient calves investigated in this study were raised separately. Dietary enteritis was diagnosed when enteric pathogens were not detected in feces during the weaning period when calves were around 2–3 months old. Weak calf syndrome was diagnosed when calves were born underweight (< 35 kg) and had low total protein in their plasma due to inadequate intake of colostrum. Blood biochemical studies were conducted at the Sanritsu Zelkova Veterinary Laboratory. In addition to the FMT treatments, 158 calves with/without diarrhea were further selected from 14 farms to identify predictive markers that could be used for future FMT donor selections as well as for successful FMT treatments.

### FMT procedure

Fresh feces (~ 100 g) were collected from healthy donors and suspended in 200–250 ml of sterilized saline. Fecal suspensions were filtered using medical gauze to remove large particles and immediately transferred intrarectally into recipients suffering from intractable diarrhea. The efficacy of FMT treatments was monitored based on physical condition, diarrheal status, and clinical status. Fecal samples were collected from donors on the day of FMT and from recipients before FMT (day 0), 1 day after FMT, and 7 days after FMT. They were directly stored at – 80 °C for metabolomics and enzyme-linked immunosorbent assay (ELISA) analyses or collected in stool nucleic acid collection and preservation tubes (Norgen) for 16S rRNA gene sequencing. Blood samples were collected from the jugular veins of recipients at days 0 and 7 for plasma analyses.

### Amplification of 16S rRNA gene by PCR

Genomic DNA was extracted from feces using a Stool DNA Isolation Kit (Norgen) according to the manufacturer’s protocol. This DNA was subjected to PCR according to previous methods [19]. The V3 and V4 regions of bacterial 16S rRNA were amplified from genomic DNA using PCR with PrimeSTAR HS DNA Polymerase (TAKARA) and the following primers: forward primers mixed (5′-TGCTCTTCCGATCTGACNNNCCTACGGGNGGCWGCAG-3′, 5′-TGCTCTTCCGATCTGACNNNNCCTACGGGNGGCWGCAG-3′, 5′-TGCTCTTCCGATCTGACNNNNNCCTACGGGNGGCWGCAG-3′, and 5′-TGCTCTTCCGATCTGACNNNNNNCCTACGGGNGGCWGCAG-3′) and reverse primers mixed (5′-CGCTCTTCCGATCTCTGNNNGACTACHVGGGTATCTAATCC-3′, 5′-CGCTCTTCCGATCTCTGNNNNGACTACHVGGGTATCTAATCC-3′, 5′-CGCTCTTCCGATCTCTGNNNNNGACTACHVGGGTATCTAATCC-3′, and 5′-CGCTCTTCCGATCTCTGNNNNNNGACTACHVGGGTATCTAATCC-3′). The adaptor tag sequence is singly underlined, whereas the spacer sequences are double underlined. The PCR fragments obtained from the first round of PCR were purified using AMPure XP and then amplified in the second round of PCR using the following primers: forward (5′-CAAGCAGAAGACGGCATACGAGATxxxxxxxxxGTGACTGGAGTTCAGACGTGTGCTCTTCCGATCTGAC-3′) and reverse (5′-AATGATACGGCGACCACCGAGATCTACACxxxxxACACTCTTTCCCTACACGACGCTCTTCCGATCTCTG-3′), which included nine and five base indices shown as “xxxxxxxxx” and “xxxxx,” respectively, to distinguish each sample. All PCR products were sequenced using the MiSeq platform (Illumina) with the MiSeq Reagent Kit v2 (500 cycles).

### Metagenomics and functional prediction analyses

Demultiplexed raw sequences were acquired from the BaseSpace Sequence Hub (Illumina), and sequences were analyzed using QIIME 2 (version 2020.2) [20]. The quality of joined sequences was filtered using the q2-demux plugin, which was followed by denoizing with DADA2 to cluster operational taxonomic units (OTUs) and generate a feature table for further analysis. The data from processed sequencing were assigned and aligned to the Greengenes reference database at 99% sequence similarity [21]. LEfSe was applied to determine the most discriminant taxa among the groups based on the feature table [22]. For statistical analyses and visual exploration, an OTU table with taxa in plain format and metadata file were analyzed using MicrobiomeAnalyst [23]. The functional potential of microbiome data from different groups was predicted based on the 16S rRNA data using Piphillin, which was established by using the Kyoto Encyclopedia of Genes and Genomes (KEGG) database and BioCyc reference database to assign functional properties [24].

### Capillary electrophoresis time-of-flight mass spectrometry metabolomics analysis

Metabolomics analysis using fecal samples was conducted at the Human Metabolome Technologies, Inc. [25]. Specifically, the samples were dried using a freeze dryer (TAITEC) and analyzed by capillary electrophoresis time-of-flight mass spectrometry (CE-TOFMS) (Agilent). The metabolite standards, instrumentation, and CE-TOFMS conditions used in this study were identical to those previously described [25]. The identified metabolites were quantified by comparing their peak areas with those of authentic standards using ChemStation software (Agilent Technologies).

### Enzyme-linked immunosorbent assay

The levels of IgA, IgM, and IgG in feces were measured using ELISA. Specifically, feces were suspended in PBS (10 μl per mg), and the supernatants were collected using centrifugation. Next, 96-well ELISA plates were coated with 500 ng/ml of sheep anti-bovine IgA (Bethyl), IgM (Bethyl), or IgG (Bethyl) overnight at 4 °C. After blocking with 0.05 % (v/v) of Tween-20 for 1 h at RT, diluted fecal suspensions were incubated in the plates for 2 h at RT. After washing, the plates were treated with 100 ng/ml of HRP-conjugated sheep anti-bovine IgA (Bethyl), IgG (Bethyl), or IgM (Bethyl) for 1 h at RT. Bovine reference serum containing IgA, IgM, and IgG with known concentrations was used as a standard.

### Weighted gene correlation network analysis

To describe the correlations of microbiomes and metabolites associated with the success or failure of FMT, coexpression networks were constructed using the weighted gene correlation network analysis (WGCNA) (v1.70-3) package in R [26]. WGCNA is a bioinformatics application in which microbial taxa form modules and link to specific traits of interest. The modules were obtained using the automatic network construction function with the default settings, except that the power was 10, TOMType was signed, and minModuleSize was 10. Modules were defined as clusters of highly interconnected microbial taxa; those taxa within the same cluster had high pairwise correlation coefficients. The modules obtained from each analysis were further analyzed in MicrobiomeAnalyst 4.0 to generate heatmaps [23].

### Multivariate data analysis

Multivariate statistical tools, including unsupervised principal component analysis (PCA) and supervised partial least squares discriminate analysis (PLS-DA), were employed to reduce and visualize the complex metabolomics datasets [27]. In PLS-DA, parameters of R2X and Q2 were used to evaluate the model quality and predictive ability, respectively. Values > 0.5 indicated that the models were robust and the predictions reliable. Score plots of the PLS-DA were applied to visualize the separation between the studied groups and loading plots were used to find candidate biomarkers responsible for the separation. These biomarkers were considered to differentiate metabolites that were selected on the basis of variable importance in projection (VIP) values > 1.0 as a threshold. *P*-values were corrected for multiple testing using a false-discovery rate (*Q*-value) method [28]. Procrustes analysis for both the microbiome and metabolome was conducted using the Procrustes function in the vegan R package.

### Statistical analysis

Alpha and beta diversity statistics were obtained using the QIIME 2 scripts diversity plugin [20]. In the calculation of alpha diversity metrics, normalization was performed using the “rarefaction” QIIME 2 process with standard parameters and by setting the max_rare_depth (upper limit of rarefaction depths) to the mean sample size. Alpha diversity metrics were calculated using Shannon’s diversity index and phylogenetic diversity. The beta diversity of the microbial profile was calculated using the QIIME 2 “diversity beta-group-significance” script. Phylogenetic (unweighted UniFrac distance) beta diversity metrics were calculated and graphically visualized by three-dimensional principal coordinate analysis (PCoA) representations and verified using a nonparametric PERMANOVA test with 999 permutations. Correlations between the selected microorganisms and metabolites were assessed by Pearson’s correlation test using “cortest” in R. To create the classifiers, RFs comprising 500 trees were computed using the default settings of the “randomForest” function implemented in the randomForest R package. All other statistical analyses were performed in Graphpad Prism version 7 (GraphPad Software).

## Results

### FMT efficacy in CD treatments

**Fig. 1:**
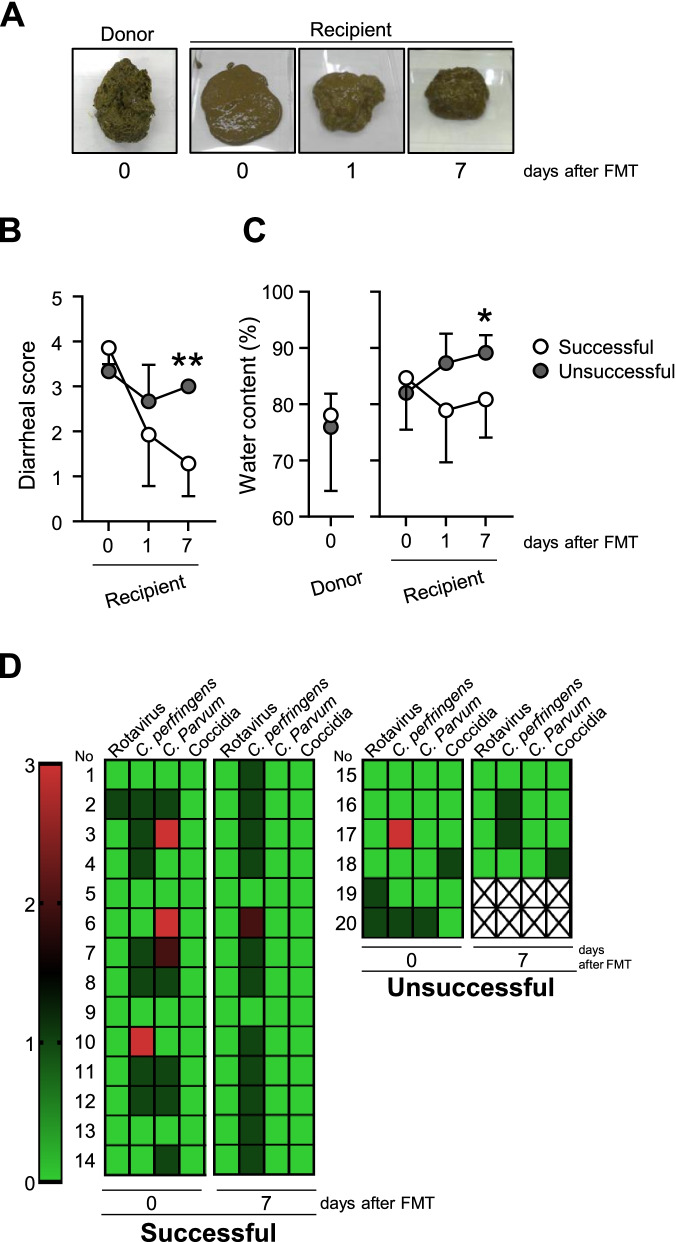
Efficacy of FMT in CD prevention. **a** Images of feces of healthy donor and recipient diarrheal calves in successful FMT treatment. **b** Diarrheal score. **c** Fecal water content. **d** FMT-specific pathogen detection. Donor (*n* = 14), recipient 0 (*n* = 14), recipient 1 (*n* = 14), and recipient 7 (*n* = 14) in successful cases; donor (*n* = 6), recipient 0 (*n* = 6), recipient 1 (*n* = 6), and recipient 7 (*n* = 4) in unsuccessful cases. 10^9^ CFU, 10^8^ CFU, and 10^7^ CFU g/feces are indicated by red, dark green, and green colors, respectively. *P < 0.05 and **P < 0.01 (Student’s *t*-test)

Twenty FMT treatments were conducted to treat recipient calves suffering from refractory CD. The efficacy of FMT treatments was determined using the diarrheal score, physical appearance, and performance from enteropathogenic microbial analysis of feces collected from the recipient calves just before and 1 week after each FMT treatment. A representative fecal sample collected from successful cases showed that the incidence of diarrhea was reduced in recipient calves (Fig. 1a). Consistent with a previous study [6], FMT was effective as a therapeutic to cure refractory CD: 14 of 20 treatments (70%) were successful in the present study (Additional file 1: Table S1). Six treatments were unsuccessful because the recipients were unable to recover from diarrhea symptoms. Among these cases, two treatments were not completed during the experiment because of unrecoverable death and shipping. Considering the age of the calves, a significant difference was observed between successful and unsuccessful recipients, but not between successful and unsuccessful donors (Additional file 1: Table S1). Importantly, these results give rise to a new challenge, i.e., identifying the essential factors responsible for not only successful but also unsuccessful FMT treatments. Indeed, the clinical distinction between successful (70%) and unsuccessful (30%) treatments was clear: diarrheal score and fecal water content decreased significantly after FMT only in successful cases (Fig. 1b, c). A classical method was employed in fecal tests to identify the causative enteropathogens: 70% (14/20) of recipients were diagnosed with infectious CD, as indicated by the presence of *C. perfringens*, *C. parvum*, rotavirus, and/or coccidia in multiple calves with diarrhea (Fig. 1d). Interestingly, *C. perfringens* was frequently detected even 7 days after the FMT treatments regardless of the symptomatologic recovery (Fig. 1d). In addition, 25% (5/20) and 5% (1/20) of calves were diagnosed with dietary enteritis and weak calf syndrome, respectively. The cure rate for dietary enteritis was 100% and 0% in successful and unsuccessful FMT treatments, respectively (Fig. S1). Along with the detection of fecal enteropathogens, blood biomarkers were assessed to monitor the efficacy of FMT treatments based on the energy metabolism, inflammation, and liver function of calves. The levels of most components did not differ between successful and unsuccessful recipients before and 7 days after FMT (Fig. S2). However, the concentration of total cholesterol was higher in successful recipients than that in unsuccessful recipients 7 days after (although not before) FMT (Fig. S2). Additionally, higher levels of γ-GT were found in successful recipients than were found in unsuccessful recipients before (but not 7 days after) FMT (Fig. S2). Overall, these results suggest that total cholesterol and γ-GT may be useful as blood biomarkers to monitor the efficacy of FMT [29, 30].

### Difference in fecal microbial composition of healthy donors and diarrheal recipients before and after FMT: successful vs unsuccessful FMT treatment

**Fig. 2:**
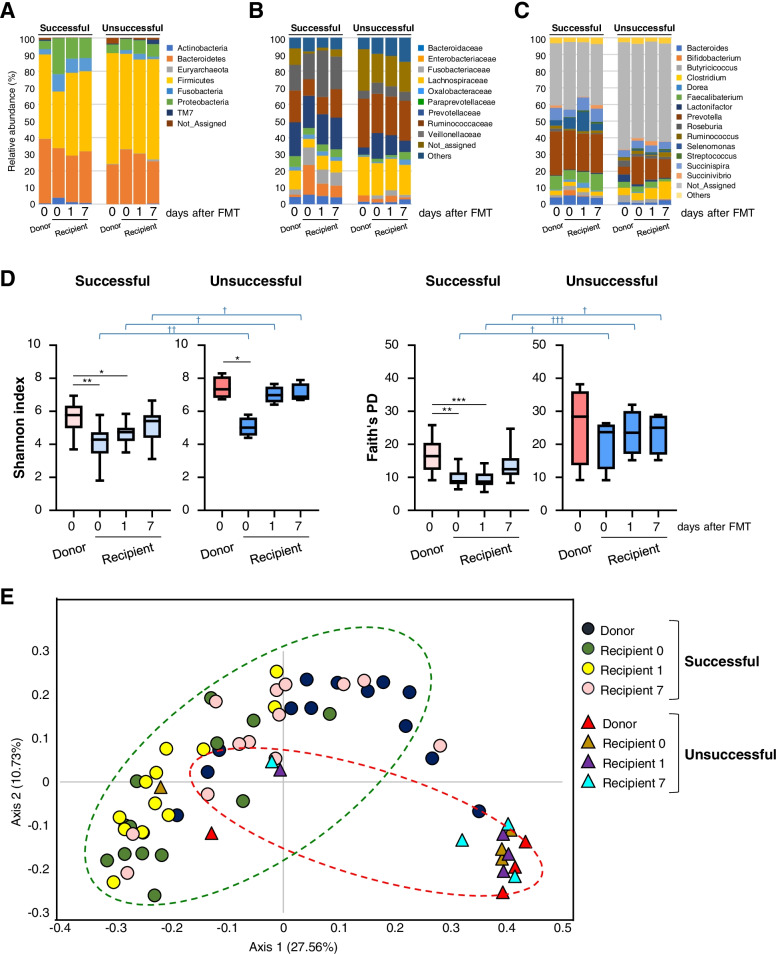
Microbial composition in the FMT study. Microbial composition at the **a** phylum, **b** family, and **c** genus levels. Donor (*n* = 13), recipient 0 (*n* = 13), recipient 1 (*n* = 13), and recipient 7 (*n* = 13) in successful FMT; donor (*n* = 4), recipient 0 (*n* = 4), recipient 1 (*n* = 4), and recipient 7 (*n* = 4) in unsuccessful FMT treatment. **d** Alpha diversity index based on Shannon’s index and faith phylogenetic diversity. **e** PCoA based on the unweighted distance matrix of the bacterial 16S rRNA gene sequence data for fecal samples (donor and recipient *n* numbers in successful and unsuccessful FMT are the same as those shown above). *P*-values (***P < 0.001, **P < 0.01, and *P < 0.05) indicate statistical significance in either successful or unsuccessful FMT treatment cases according to one-way ANOVA followed by Tukey’s multiple comparison test. *P*-values (^†††^*P* < 0.001, ^††^*P* < 0.01 and ^†^*P* < 0.05) indicate statistical significance according to Student’s *t*-test

The microbial compositional difference between successful and unsuccessful FMT treatments was first assessed using 16S rRNA gene sequencing; high-quality sequences were clustered into OTUs according to a cutoff of 97% sequence similarity using the QIIME2 bioinformatics platform [20]. Analysis at the phylum level did not show a clear difference between successful and unsuccessful FMT treatments (Fig. 2a; Fig. S3). Microbial community analysis at the family level showed that *Veillonellaceae* was found in higher numbers in successful cases, whereas *Lachnospiraceae*, *Ruminococcaceae*, *Methanobacteriaceae*, *Peptostreptococcaceae*, *Odoribacteraceae*, and *Barnesiellaceae* were found in higher numbers in unsuccessful cases (Fig. 2b; Fig. S4). According to the genus level analysis, the abundance of *Clostridium* and *Methanobrevibacter* was significantly higher after FMT in unsuccessful cases (Fig. 2c; Fig. S5). Alpha diversity analysis [31] was conducted using the Shannon index and phylogenetic diversity (Faith’s PD) in QIIME2; the results showed that more diverse and distinct bacterial communities were present in donors compared with the bacterial communities in recipients with diarrhea before FMT treatments that were both successful and unsuccessful (Fig. 2d). In the recipients from both cases, alpha diversity indexes tended to increase after FMT (Fig. 2d). Beta diversity analysis was conducted using nonparametric permutational multivariate ANOVA (PERMANOVA) tests with 999 permutations to measure the compositional similarities between bacterial communities within groups of samples. For this, abundance data based on unweighted UniFrac distance matrices were analyzed; the results showed that significant divergences existed between the groups (Fig. 2e). Specifically, in successful FMT treatments, statistical differences in the distance were observed between donors (D-success) and recipients just before FMT (R-0-success) and between D-success and recipients 1 day after FMT (R-1-success), but not between D-success and recipients 7 days after FMT (R-7-success) (Additional file 1: Table S2). In contrast, in unsuccessful FMT treatments, there were no significant differences between D-failure and R-0-failure, D-failure and R-1-failure, or D-failure and R-7-failure (Additional file 1: Table S2). Thus, in successful, but not in unsuccessful cases, recipient calves gained a healthy donor microbiome composition and showed signs of donor–recipient engraftment in their gastrointestinal tract at day 7.

### Identification of the microorganisms associated with success and failure of FMT in donors and recipients

**Fig. 3:**
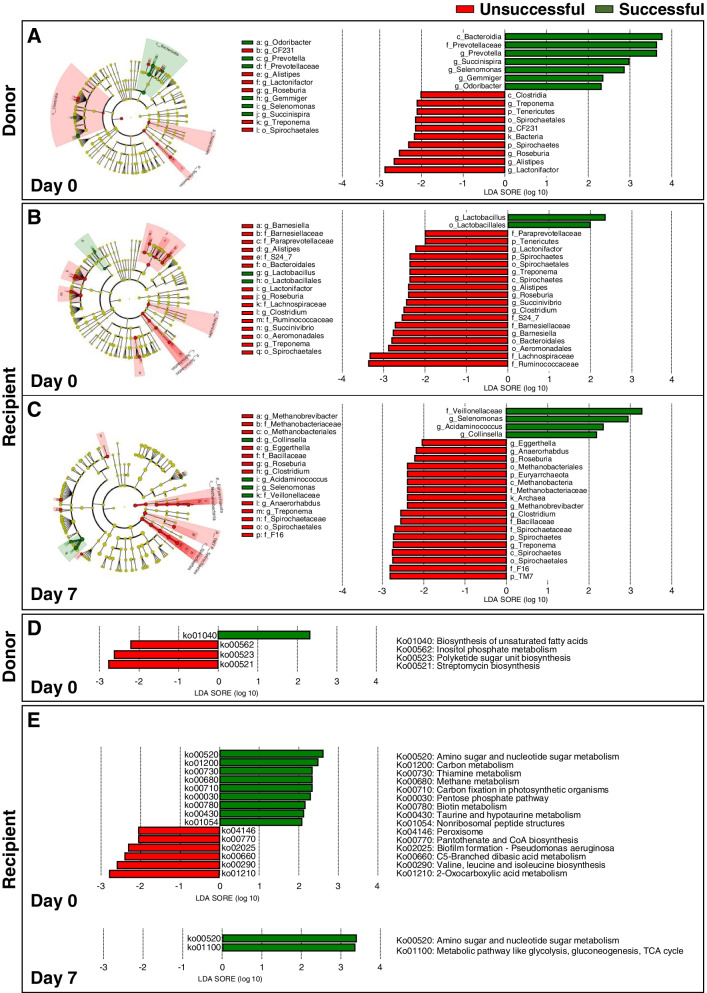
Identification of abundant microbiota. **a**–**c** Cladogram [phylum (p), class (c), order (o), family (f), genera (g)] and linear discriminant analysis (LDA) scores of abundant taxa in the donor (day 0) (**a**), recipient day 0 (**b**), and recipient day 7 (**c**) [donor (*n* = 13), recipient 0 (*n* = 13), recipient 1 (*n* = 13), and recipient 7 (*n* = 13) in successful FMT; donor (*n* = 4), recipient 0 (*n* = 4), recipient 1 (*n* = 4), and for recipient 7 (*n* = 4) in unsuccessful FMT]. Predicted KEGG metabolic pathways related to successful or unsuccessful FMT treatment cases in donors (**d**) and recipients (**e**) in identified by Piphillin and LDA (LDA > 2.0, *P* < 0.05). Data shown for successful and unsuccessful FMT treatments for donors and recipients (*n* numbers are the same as those shown above)

We hypothesized that, in successful FMT treatments, FMT-induced remission of diarrhea might be the result of a commensal bacterial community being generated and CD-causative pathogens being eradicated. To contextualize our findings, linear discriminant analysis effect size (LEfSe) [22] was conducted to investigate the differential abundance of microbial taxa between successful and unsuccessful FMT treatments in donors and recipients. When donors were compared by the success or failure of FMT, the family *Prevotellaceae* and genera *Prevotella*, *Succinispira*, and *Selenomonas* were higher relative abundance in the successful FMT treatments, whereas the genera *Lactonifactor*, *Alistipes*, and *Roseburia* were found at the higher relative abundance in the unsuccessful FMT treatments (Fig. 3a). Discriminatory taxa were not identified, however, in either successful or unsuccessful recipients 1 day after FMT. In successful recipients, the genus *Lactobacillus* showed increased differential abundance prior to FMT (R-0-success) at day 0, whereas the family *Veillonellaceae* and genera *Selenomonas*, *Acidaminococcus*, and *Collinsella* were significantly more abundant 7 days after FMT (R-7-success) (Fig. 3b, c). In unsuccessful recipients, multiple bacteria were identified as discriminatory taxa prior to FMT (R-0-failure) and 7 days after FMT (R-7-failure). Among these discriminatory taxa, the phyla *Tenericutes* and *Spirochaetes* were also found in unsuccessful donors (D-0-failure) (Fig. 3a, b). It should be emphasized that the genus *Selenomonas* was also found in successful donors (D-0-success), suggesting that it may act as a signature microbe and could have the potential to ensure donor–recipient compatibility (Fig. 3a, c). Moreover, further analysis using Piphillin [24], an algorithm that can be applied to interpret the potential functions of a microbial community, identified 264 KEGG pathway modules as FMT-related pathways (Additional file 2: Dataset S1). Subsequent LEfSe analysis based on the results from Piphillin revealed that the pathways ko01040 (biosynthesis of unsaturated fatty acids) and ko00521 (streptomycin biosynthesis) were enriched in successful and unsuccessful donors, respectively (Fig. 3d). Pathways ko00520 (amino sugar and nucleotide sugar metabolism) and ko01210 (2-oxocarboxylic acid metabolism) were significantly enriched before FMT (day 0) in successful and unsuccessful recipients, respectively, whereas ko00520 and ko01100 (Glycolysis, gluconeogenesis, TCA cycle) were identified as enriched metabolic pathways activated 7 days after FMT in successful recipients (R-7-success) (Fig. 3e).

### Difference in the fecal metabolite composition of healthy donors and diarrheal recipients before and after FMT: successful vs unsuccessful FMT treatments

**Fig. 4:**
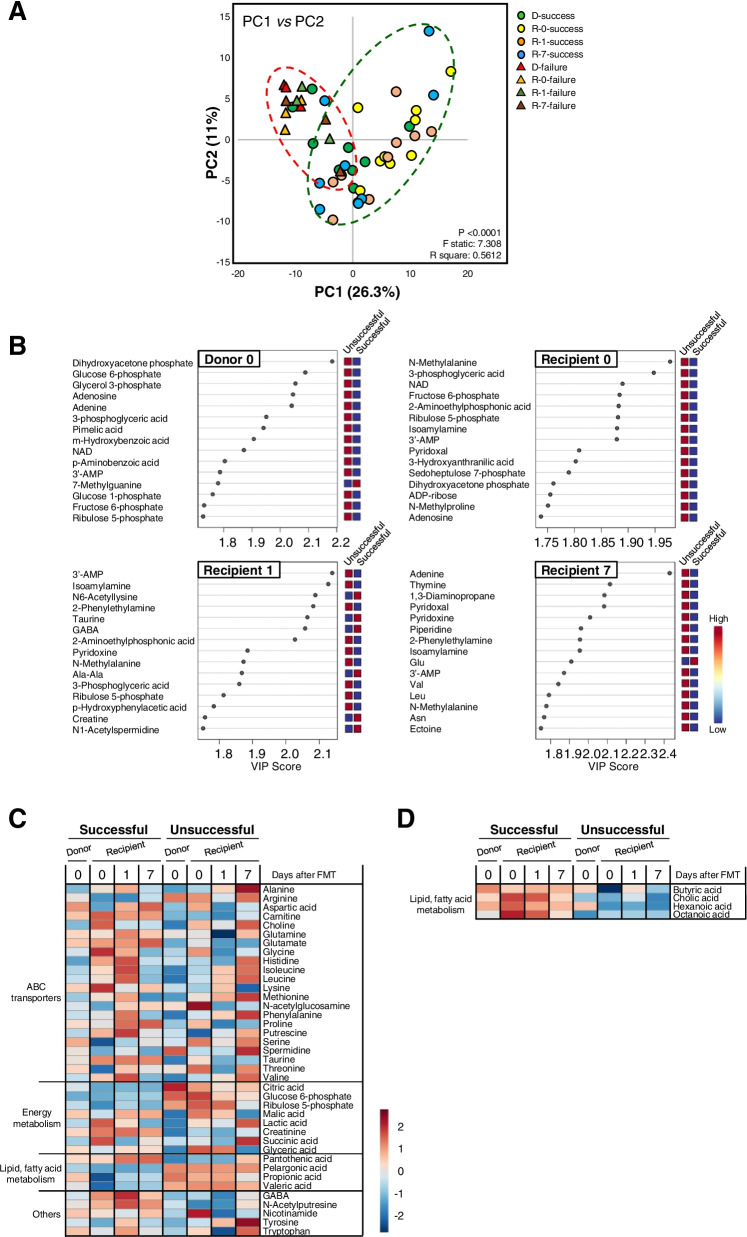
Multivariate unsupervised principal component analysis for the metabolomes of the calves’ fecal samples. Data were obtained using CE-TOFMS analysis for donor (*n* = 9), recipient 0 (*n* = 9), recipient 1 (*n* = 9), and recipient 7 (*n* = 9) in successful FMT treatment, and for donor (*n* = 3), recipient 0 (*n* = 3), recipient 1 (*n* = 3), and recipient 7 (*n* = 3) in unsuccessful FMT treatment. **a** Fecal metabolomics analysis to identify potential biomarkers. **b** Variable importance in projection (VIP) scores obtained from the partial least squares discriminant analysis for donors (at day 0) and recipients (on days 0, 1, and 7). **c**, **d** Heatmaps based on the group-based relative concentration of the major metabolites responsible for ABC transporters, lipid and fatty acid metabolism, sugar metabolism, and others in both successful and unsuccessful FMT treatment cases. Major metabolites for lipid and fatty acid metabolism are based on relative area. Each colored cell on the map corresponds to a concentration value, with samples shown in rows and metabolites in columns (donor and recipient *n* numbers in successful and unsuccessful FMT treatment cases are the same as those shown above)

To investigate the effects of FMT-induced changes in the gut microbiome on intestinal metabolism, most fecal samples collected from 12 FMT treatments (9 successful cases; 3 unsuccessful cases) were analyzed using CE-TOFMS. In total, 366 peaks composed of cations (214 peaks) and anions (152 peaks), and 264 peaks, including 159 cations and 105 anions, were attributable to known standard metabolites that could be quantified (Fig S6). Consistent with the microbial composition, PCA indicated that data points were widely dispersed on plots of fecal metabolomes in successful and unsuccessful FMT treatments (Fig. 4a). The distance among the groups based on PC1 scores is illustrated in Additional file 1: Table S3. Interestingly, PLS-DA showed that there were compositional differences in the metabolites of both donors and recipients between the treatments (Fig. S7). Specifically, when comparing successful and unsuccessful donors on the day of FMT (D-0-success vs D-0-failure), 65 potential metabolites with a VIP score [32] > 1 were identified from the PLS-DA model (Additional file 2: Dataset S2); the top 15 metabolites, including dihydroxyacetone phosphate, glucose 6-phosphate, and glycerol 3-phosphate, are shown in Fig. 4b. By comparing the successful and unsuccessful recipients, 85, 74, and 73 metabolites with VIP scores > 1 were identified following analysis prior to FMT (R-0-success vs R-0-failure), 1 day after FMT (R-1-success vs R-1-failure), and 7 days after FMT (R-7-success vs R-7-failure), respectively (Additional file 2: Dataset S3–5). The top 15 metabolites are shown for each analysis in Fig. 4b. Furthermore, changes in the major metabolites of adenosine triphosphate (ATP)-binding-cassette (ABC) transporters, lipid and fatty acid metabolism, and sugar metabolism were investigated (Fig. 4c; Figs. S8 and S9). In addition, other compounds responsible for lipid and fatty acid metabolism were identified based on their relative area due to the lack of standards available (Fig. 4d; Fig. S10). In successful but not in unsuccessful recipients, amino acid metabolism was high prior to FMT (R-0-success) and 1 day after FMT (R-1-success) compared with that observed 7 days after FMT (R-7-success). Specifically, glucogenic amino acids (alanine, aspartate, glutamine, glutamic acid, methionine, proline, serine, threonine, and valine), glucogenic and ketogenic amino acids (phenylalanine and tyrosine), and ketogenic amino acids (leucine and lysine) differed significantly among these groups (Fig. S8). The polyamines spermidine and putrescine, and another diamine, cadaverine, were also elevated in successful cases (Fig. S11). These results suggest that successful FMT treatments may be accompanied by changes in metabolites and especially by reduced concentrations of amino acids related to FMT-induced changes in gut microbiota.

### Microbiome data correlate with metabolite profiles in successful but not unsuccessful treatments

**Fig. 5:**
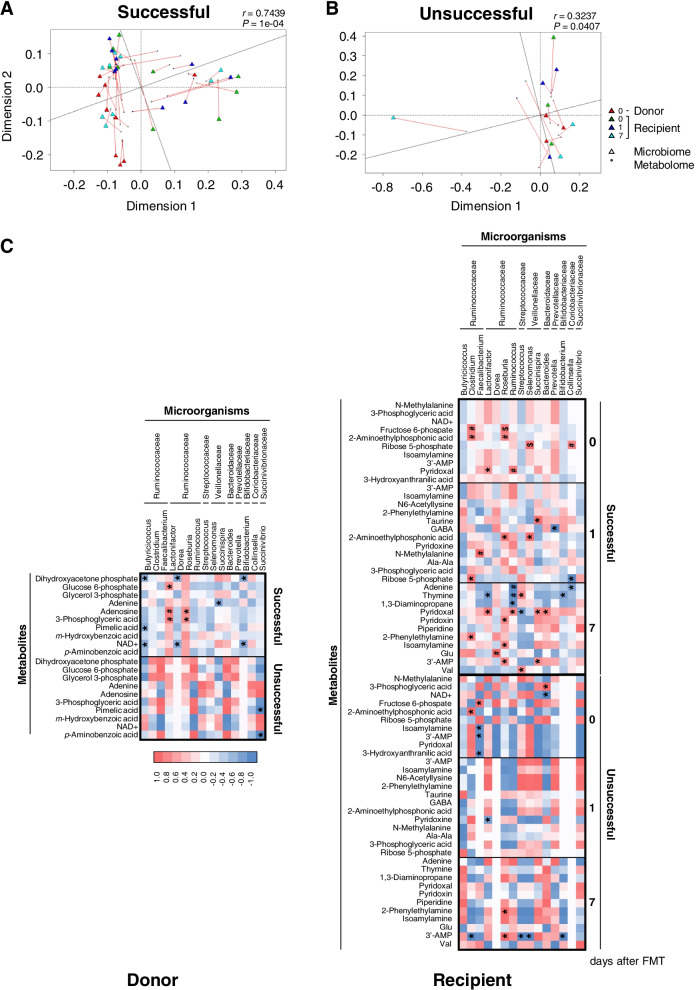
Procrustes analysis for correlations between microbiota and metabolites. Longer lines on the Procrustes plot specify more within-subject dissimilarity. **a** Overall successful FMT treatment (Procrustes sum of squares, 0.4467; correlation, 0.7439; *P* = 0.0001). **b** Overall unsuccessful FMT treatment (Procrustes sum of squares, 0.611; correlation, 0.6237; *P* = 0.0407). Data are shown for donor (*n* = 9), recipient 0 (*n* = 9), recipient 1 (*n* = 9), and recipient 7 (*n* = 9) in successful cases, and for donor (*n* = 3), recipient 0 (*n* = 3), recipient 1 (*n* = 3), and recipient 7 (*n* = 3) in unsuccessful FMT treatment cases. **c** Pearson correlations in donor (D-success and D-failure) and **b** for the recipient group at days 0, 1, and 7 (donor and recipient *n* numbers, as shown above for successful and unsuccessful FMT treatment). Color shows Pearson correlation coefficient distribution: blue represents a significant negative correlation (*P* < 0.05), red represents a significant positive correlation (*P* < 0.05), and white represents a non-significant correlation (*P* ≥ 0.05). ^$^*P* < 0.001, ^#^*P* < 0.01, and *P < 0.05 indicate statistical significance

To investigate microbiota–metabolite correlations in successful and unsuccessful FMT treatments, Procrustes analysis [33] and the Mantel test were performed using the vegan package in R [34]. Procrustes analysis of Euclidean distances between metabolomes and unweighted UniFrac distances highlighted the significant association between the microbiota taxonomic and metabolic profiles. Specifically, significant relatedness (Procrustes correlation = 0.7439, *P* = 0.0001) between the microbiota and metabolites was observed in successful cases (Fig. 5a), whereas a relatively low correlation (Procrustes correlation = 0.3237, *P* = 0.0407) was observed in unsuccessful cases (Fig. 5b). In addition, group-specific Procrustes analyses aimed at distinguishing between successful and unsuccessful FMT, donors and recipients, and results before and after FMT in recipients showed that a correlation was only observed in successful donors (D-0-success, *P* = 0.0037) and successful recipients 7 days after FMT (R-7-success, *P* = 0.0022) (Fig. S12A-H). Furthermore, the functional correlation between alternations in the microbiota and metabolites was assessed using Pearson correlation analysis based on 14 potential bacterial genera (shown in Fig. 2c) and metabolites (with VIP scores > 1.8; shown in Fig. 4b) that could have contributed substantially to the differences between the groups. In donors, *Lactonifactor* and *Roseburia* were positively correlated with the presence of 3-phosphoglysercic acid (a major compound in glycolysis) in successful cases, whereas *Succinivibrio* was negatively correlated with the presence of pimelic acid and P-aminobenzoic acid in unsuccessful cases (Fig. 5c). In recipients, prior to FMT, *Clostridium* and *Roseburia* were positively correlated with the presence of fructose 6-phosphate and 2-aminoethylphosphonic acid in successful cases (R-0-success), whereas *Clostridium*, but not *Roseburia*, was positively correlated with the presence of 2-aminoethylphosphonic acid in unsuccessful cases (R-0-failure) (Fig. 5c). In recipients 1 day after FMT, *Succinispira* was positively correlated with the occurrence of taurine, which is linked to primary bile acid biosynthesis and ABC transporters, in successful recipients (R-1-success) (Fig. 5c). *Clostridium* and *Butyricicoccus* were positively correlated with the detection of ribose 5-phosphate regardless of the success (R-1-success) or failure (R-1-failure) of FMT. In recipients, 7 days after FMT, *Ruminococcus* was negatively correlated with the presence of adenine, thymine, and 1,3-diaminoporopane in successful cases (R-7-success), but it was positively correlated with the presence of adenine in unsuccessful cases (R-7-failure) (Fig. 5c). Although metabolites were correlated with the changes in bacterial taxa, these results suggest that interactions may differ between successful and unsuccessful FMT treatments.

### WGCNA of FMT outcome

**Fig. 6:**
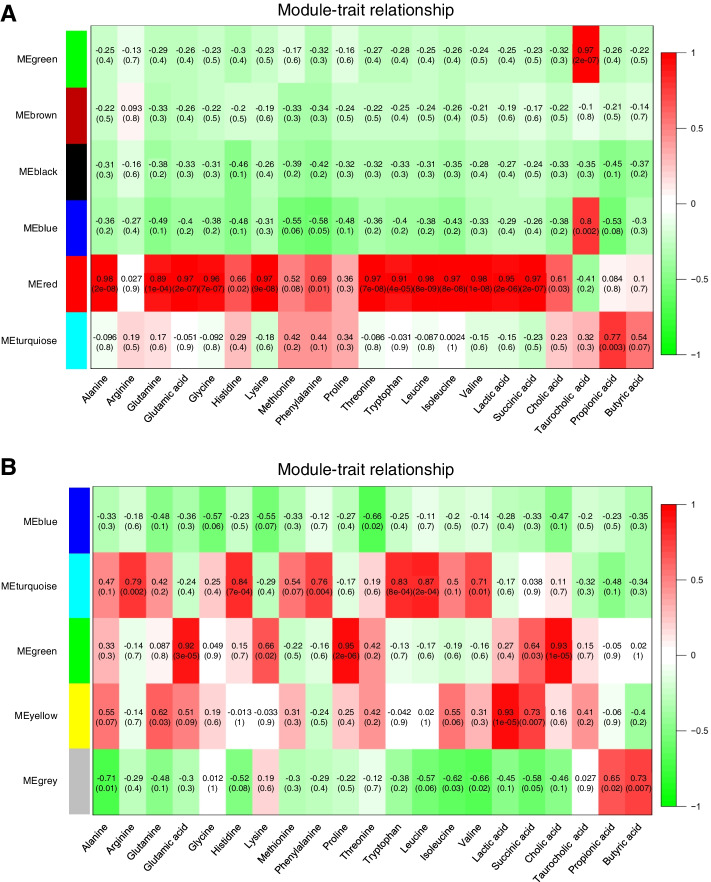
Weighted gene coexpression network analysis for microbiota and metabolites. Module-trait associations based on **a** donor groups for donors (*n* = 9) in successful FMT and donors (*n* = 3) in unsuccessful FMT treatment cases. **b** Recipients at day 7 for recipients (*n* = 9) in successful FMT and recipients (*n* = 3) in unsuccessful cases. Each row corresponds to a module. Each column corresponds to a phenotypic trait (labeled below column). Each cell at the row–column intersection contains the correlation coefficient and *P*-values (in brackets) for the module and the corresponding trait. A highly positive correlation between a specific module and a trait is indicated by dark red, whereas a negative correlation is indicated by green

If FMT is to be applied as a potential therapeutic for CD prevention, it is important to understand how microbiota and microbial products affect the incidence of CD as well as the success or failure rate of FMT. Therefore, WGCNA [26] was performed to detect the possible inherent association among microbial taxa, clinical traits, and metabolites in both donors and recipients (Additional file 3). Major microbiota found in successful FMT treatments, e.g., *Collinsella*, *Prevotella*, *Gemmiger*, *Acidaminococcus*, and *Selenomonas* (Fig. 3), were mostly found in the gray module, which implies that this module is associated with FMT success. The microbial taxa responsible for each module are shown in Fig. S13 and Additional file 2: Dataset S6. Concentrations of amino acids and organic acids were affected by both donor and recipient groups before and after FMT in both successful and unsuccessful FMT treatments (Fig. 4c, d); thus, WGCNA was individually extended to donors and recipients (days 0, 1, and 7) based on the selected amino acids, major metabolites in the TCA cycle, bile acids, and SCFAs. In donors, from the coexpression modules significantly associated with traits, modules 4 of 6 comprised taxa that were associated with several traits, e.g., amino acid-, lactic acid-, and succinic acid-related module (MEred); taurocholic acid-related module (MEblue and MEgreen); and propionic acid-related module (MEturquoise). Major microbial taxa linked to the D-success group, especially *Prevotella*, *Selenomonas*, *Succinispira*, and *Odoribacter*, were positively correlated with the MEturquoise module (Fig. 6a; Fig. S14, and Additional file 2: Dataset S7). For recipients, at day 0, four modules were formed based on the microbial taxa and traits of interest. MEblue was positively correlated with the presence of alanine, glycine, and cholic acid (|*r*| ≥ 0.5, *P* <0.05). The trait for taurocholic acid was associated with MEblue and MEturquoise (|*r*| ≥ 0.5, *P* <0.05) (Fig. S15A-B and Additional file 2: Dataset S8). At day 1, microbial taxa were categorized into six modules (Fig. S16A; Additional file 2: Dataset S9). Specifically, the genera *Selenomonas* and *Acidaminococcus* were positively correlated with MEbrown; for MEturquoise, the genus *Sporobacter* was positively correlated with the detection of succinic acid (|*r*| ≥ 0.5, *P* < 0.05) (Fig. S16B). At day 7, microbial taxa were categorized into five modules (Fig. 6b; Fig. S17 and Additional file 2: Dataset S10). In MEyellow, microbes such as *Selenomonas*, *Lactobacillus*, and *Acidaminococcus*, which were found in the R-7-success group (Fig. 3c), showed positive correlations with the occurrence of lactic acid and succinic acid (Fig. 6b; Fig. S17). In contrast, *Methanobrevibacter*, *Eggerthella*, and *Clostridium*, all of which were linked to R-7-failure (Fig. 4; Fig. S17), were significantly correlated with MEturquoise, which included the amino acids arginine, histidine, leucine, and phenylalanine (Fig. 6b). Thus, these microbial taxa could potentially be considered predictive biomarkers of FMT failure in CD prevention.

### Sporobacter is a potential biomarker of appropriate donors for FMT

**Fig. 7:**
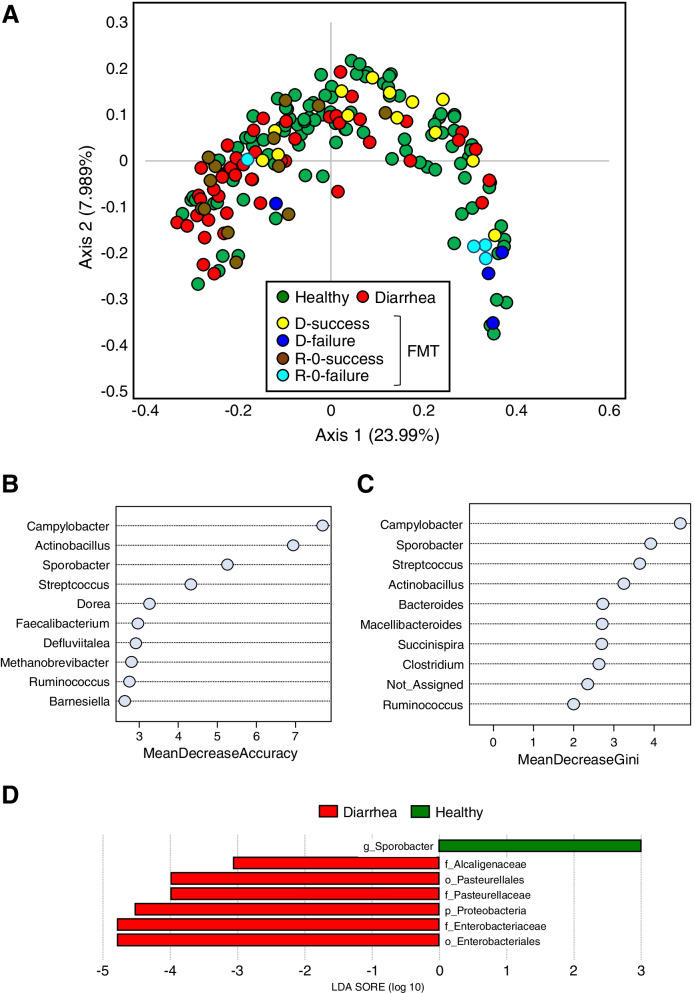
Rational donor selection for successful FMT prediction. **a** PCoA based on unweighted UniFrac distance of overall healthy and diarrheal calves used in FMT. Random-forest classification based on **b** the mean decrease accuracy and **c** the mean decrease gini. **d** Linear discriminant analysis (LDA) score obtained from linear discriminant analysis shown for healthy (*n* = 105), diarrheal (*n* = 46), D-success (*n* = 13) and R-0-success (*n* = 13) from successful FMT treatment cases, and D-failure (*n* = 4) and R-0-failure (*n* = 13) from unsuccessful FMT treatment cases

To select optimal donors and diarrheal recipients for FMT, 158 diarrhea and non-diarrhea fecal samples were collected (Additional file 1: Table S4), and then 16S rRNA gene amplicon sequencing was performed to compare calves defined as donors and recipients in either successful or unsuccessful FMT treatments. Diarrheal scores and microbial compositions at the phylum level are given in Fig. S18A, B. To select the potential predicators, e.g., specific microorganisms, for successful cases, a PCoA of the unweighted UniFrac distance matrix was performed on healthy and diarrheal calves (Fig. 7a). A significant difference was observed between healthy calves and unsuccessful, but not successful, donors in UniFrac distance analysis (Additional file 1: Table S5), suggesting that the donors selected for FMT may be inappropriate in unsuccessful FMT treatments. Finally, an RF model was constructed to identify potential biomarkers in the overall healthy and diarrheal calves along with calves recruited for FMT [35]. *Campylobacter*, *Actinobacillus*, and *Sporobacter* were identified based on the mean decrease in accuracy; *Campylobactor*, *Sporobacter*, and *Streptococcus* were identified as the most discriminating predictors based on the mean decrease in the gini criteria (Fig. 7b, c). Considering microbial abundance, *Sporobacter* was abundant in the overall healthy and donor groups from successful FMT (Fig S18C). In contrast, *Camphylobacter* was found abundantly in the recipient diarrheal group following unsuccessful FMT treatments (Fig. S18D). Along with RF, LEfSe analysis was subsequently performed among these groups, in which *Sporobacter* was found to be abundant in the healthy group (Fig. 7d). Taken together, these results suggest that *Sporobacter* may be a potential biomarker for the donors associated with FMT success.

## Discussion

The success rate of FMT as a treatment for recurrent *C. difficile* infection in humans is extremely high at > 80%, i.e., patients do not develop diarrhea and/or *C. difficile* is not detected in their feces at any time for 8 weeks post-treatment [36]. In a recent study, oral FMT was shown to be effective at treating multifactorial CD in multiple doses compared with classical therapeutics such as antibiotics [6]. As CD has a substantial negative impact on the livestock industry, determining the efficacy of FMT in CD prevention and establishing the procedure as a practical therapeutic option is a priority. The aforementioned study [6] followed up the efficacy of FMT for 48 days; however, farmers have a notable tendency to overcome CD rapidly from a commercial farm management perspective. Therefore, we focused on the changes in the intestinal environment of diarrheic calves during the first 7 days following a single rectal FMT. Consistent with the previous study [6], 70% of our FMT treatments were successful, as shown by the dramatic decrease in diarrheal scores. In additional analyses, we considered the 30% of unsuccessful FMT treatments with no changes in diarrheal score in an effort to identify the essential factors of FMT efficacy. According to the reductions in dissimilarity between donors and recipients identified by comparing the unweighted UniFrac distance in beta diversity analysis, no baseline difference was observed. However, when considering the alpha diversity index, both the Shannon index (an indicator of richness and evenness) and Faith’s PD (an indicator of phylogenic distance) for successful FMT treatments were found to differ significantly in donors and recipients before but not after FMT. In unsuccessful FMT cases, only the Shannon index but not Faith’s PD was significantly different in donors and recipients before FMT. These data indicate that a baseline difference existed between donors and recipients in unsuccessful FMT treatments. However, the lack of unique OTUs (based on Faith’s PD) in unsuccessful donors may have failed to confer the microbial changes in the recipient’s gut microbiota composition. Therefore, it is possible that FMT treatments could be unsuccessful when the microbiome is similar between the recipients and donors.

In this study, an important advance was the discovery of beneficial and nonbeneficial bacteria identified from successful and unsuccessful FMT treatments. The genus *Selenomonas*, which utilizes lactic acid [37], could be transferred from donors to recipients in successful (but not unsuccessful) cases within 7 days after FMT treatments. The presence of *Selenomonas* in donors and recipients that have recovered from diarrhea is thought to be important for enhancing energy metabolism; several species of *Selenomonas* are known to be involved in ATP production through their effects on the succinate–propionate pathway [38]. WGCNA also confirmed the importance of *Selenomonas*, which was positively correlated with the amounts of lactic acid and succinic acid in donors and recipients 1 and 7 days after FMT in successful (but not unsuccessful) FMT treatments. Another important discovery was that of the genus *Lactobacillus*, which was abundant in recipients prior to FMT in successful (but not unsuccessful) cases. *Lactobacillus* promotes the growth of commensal microbiota and may participate in a mutualistic relationship between the host and microbes in the gastrointestinal tract of successful recipients after FMT [39]. In analysis at the species or strain levels, *Lactobacillus rhamnosus* CRL1505 has been known to modulate antiviral immune responses in intestinal epithelial cells and antigen-presenting cells (APCs), such as macrophages and dendritic cells, to increase resistance against intestinal viral infections [40]. *Lactobacillus rhamnosus* CRL1505 also augments the Th1 response triggered by TLR2 signaling and increases the expression of MHC class II molecules as well as IL-1β, IL-6, IL-10, and IFN-γ in APCs for modulating intestinal immunity and thereby improving immune-health status [40]. In contrast, the phylum *Spirochaetes*, which is responsible for mucohaemorrhagic colitis in swine [41], was abundant in unsuccessful donors and in unsuccessful recipients throughout the experiment even after FMT. Further studies are required to explore the role of *Spirochaetes* in the occurrence or progression of CD; nevertheless, these results suggest that this phylum may negatively affect the efficacy of FMT. Overall, *Selenomonas*, *Lactobacillus*, and *Spirochaetes* seem to be microorganisms associated with donors and/or recipients that lead to the success or failure of FMT; thus, a convenient strategy for detecting the presence of such bacteria in the field should be established to increase the rate of FMT success.

This study also confirmed dynamic changes in metabolites belonging to successful and unsuccessful FMT treatments. In successful cases, short-chain fatty acids, especially butyric acid, and medium-chain fatty acids, such as octanoic acid, were enriched after FMT treatments. The genus *Collinsella* is capable of producing butyric acid [42]; consistently, *Collinsella* and butyric acid were both discriminately abundant 7 days after successful FMT treatments. Therefore, the intestinal microbiota appears to coevolve with the host and play a crucial role in host nutrient absorption and metabolism [43]. Furthermore, in unsuccessful cases, FMT treatment upregulated the excretion of metabolites that are members of the ABC transport system superfamily, which contains amino acids, choline, putrescine, and spermidine. Given that ABC transporters are involved in the transport of a variety of substances ranging from simple ions to large molecules as well as signal transduction, drug and antibiotic resistance, and antigen presentation [7, 44], it is possible that these transporters function abnormally in unsuccessful cases. The gene expression associated with ribosomal translation, amino acid metabolism, and carbohydrate metabolism is reduced in calves suffering from hemorrhagic diarrhea [3, 6, 13]; therefore, in additional FMT studies, it will be important to identify the key microbial clusters that regulate intestinal metabolites. Altered amino acid metabolism has also been observed in cats and dogs suffering from chronic diarrhea [45, 46]; consequently, amino acid changes may be a factor in the remission of CD. Consistent with a previous study [6], we observed decreased levels of most fecal amino acids in the successful recipients 7 days after FMT (compared with the original levels prior to therapy). In contrast, the fecal amino acid levels mostly increased after FMT in unsuccessful recipients. Furthermore, the significant microbial taxa of the R-7-failure group (e.g., the family *Bacillaceae* and genera *Methanobrevibacter*, *Eggerthella*, and *Clostridium*) were positively correlated with the levels of branched-chain amino acids (i.e., leucine and valine) as well as with levels of arginine, histidine, phenylalanine, and tryptophan. Given that the amino acids present in feces have been positively correlated with the severity of Crohn’s disease [47], the high amounts of free amino acids detected in unsuccessful FMT cases suggest that suboptimal nutrient utilization occurred due to dysbiosis. Furthermore, the metabolic milieu generated during unsuccessful FMT treatment may help to regulate the colonization and pathogenicity of harmful microbiota. For example, biogenic amino acids, such as isoamylamine, were identified as potential markers in unsuccessful FMT treatments. Previously, isoamylamine was shown to play a specific role in the encystation and differentiation processes of pathogenic protozoa, e.g., *Entamoeba*, *Giardia*, *Acanthamoeba*, and *Balamuthia*, and to exhibit a distinct kinetic feature of their survival [48]. Dihydroxyacetone phosphate and glycerol-3-phosphate, which had the highest VIP scores in unsuccessful donors in the present study, are dominantly produced by the flagellated protozoan *Giardia duodenalisis*, a worldwide parasite that causes giardiasis (an acute and chronic diarrheal disease) [49]. To ensure the success of FMT, it may be necessary, therefore, to also ensure the absence of metabolites associated with unsuccessful FMT in both donors and recipients prior to treatment.

Notably, the establishment of FMT as a veterinary practice for the effective treatment of CD remains difficult due to safety concerns regarding the transmission of virulence factors [50]. It is also difficult to determine the potential for success from donors prior to FMT; however, a machine learning approach has provided some predictions of the microbial composition required in potential donors [51]. We identified *Sporobacter* as a potential biomarker for donor selection using RF classification and LEfSe analysis; *Sporobacter* was also positively correlated with the production of succinic acid in R-1-success, suggesting that it may play an important role in the interactions of microbiota and metabolites in potential donors. Establishing discriminative characteristics using the novel prediction model applied in this study to identify calves with diarrheal incidence that were at high risk of failure even after FMT therapy may also be useful for veterinary physicians in relation to their management plans. Thus, our study emphasizes the use of a machine learning approach to help protect livestock. Further studies are needed to determine whether the donor FMT itself induces the complete resolution of CD or whether it helps promote a spontaneous recovery. Indeed, it will be important to develop a robust understanding of the gut microbiome and metabolome across individuals in general.

This study was the first to examine multidonor FMT as a strategy for CD treatment through the remediation of gut microbiota with an emphasis on the personalization of interventions; additionally, the inherent individualized variability in microbiota, metabolites, and other physiological features was considered. Our findings provide new insights into developing successful FMT strategies with a multiomics approach, and they may help advance research into microbial therapeutics for CD treatment.

## Conclusion

In this study, we demonstrated that FMT is an effective treatment option for the prevention of diarrhea in calves, specifically by identifying a beneficial microbial cluster as well as functional metabolites. We showed that FMT success or failure depends on the microbial composition of both donors and recipients. We also consistently identified the microbial genera *Sporobacter* and *Selenomonas* in donor calves and *Lactobacillus* in recipient diarrheal calves in successful FMT cases; thus, the presence of these genera may predict FMT success. These findings have enormous significance for the livestock industry because FMT could eventually address the challenge of CD prevention as well as the use of excessive antibiotics.

## Supplementary Information

**Additional file 1: Table S1.** Demographic information of the calves selected for FMT. **Table S2.** Unweighted distance matrix among groups. **Table S3.** P values of the Tukey’s multiple comparison test from the one-way ANOVA between PC1 scores of the PCA. **Table S4.** Demographic information of the selected healthy and diarrheal calves. **Table S5.** Unweighted unifrac distance matrix analysis.

**Additional file 2.** Datasets 1-10.

**Additional file 3.** Module; trait.

**Additional file 4: Fig. S1.** Overview of farm specific cow and disease condition. The effect of FMT on enteritis and weak calf syndrome disease prevention in successful and unsuccessful treatments. (A) Farm specific cows’ number. (B) Success rate evaluated by presence and absence of enteritis and weak calf syndrome before and after FMT for recipient (*n* = 14) in successful treatments and recipient (*n* = 6) in unsuccessful treatments. ‘+, presence; ‘-, absence. **Fig. S2.** Clinical parameter. Clinical efficacy of FMT on successful and unsuccessful treatments based on day-0 (before FMT) and day-7 (after FMT). Data are expressed as mean±SD, shown for R-0-success (*n* = 14), R-0-failure (*n* = 6), R-7-success (*n* = 14), and R-7-failure (*n* = 4). γ-GT, Gamma-Glutamyl Transferase; MCV, mean corpuscular volume; MCH, mean corpuscular hemoglobin; MCHC, mean corpuscular hemoglobin concentration. *P <0.05 (Student’s *t*-test). **Fig. S3.** Microbial composition based on phylum level. Relative abundances are shown as mean ± SEM for donor (*n* = 13), recipient 0 (*n* = 13), recipient 1 (*n* = 13), and recipient 7 (*n* = 13) in successful treatments, and donor (*n* = 4), recipient 0 (*n* = 4), recipient 1 (*n* = 4), and for recipient 7 (*n* = 4) in unsuccessful treatments. *P*-values (*P <0.05, **P <0.01) indicate statistical significance either successful or unsuccessful treatments by one-way ANOVA followed by tukey multiple comparison test. *P*-values (^††^*P* <0.01, ^†^*P* <0.05) indicate statistical significance by Student’s *t*-test. **Fig. S4.** Microbial composition based on family level. Relative abundances are shown as mean ± SEM for donor (*n* = 13), recipient 0 (*n* = 13), recipient 1 (*n* = 13), and recipient 7 (*n* = 13) in successful treatments, and donor (*n* = 4), recipient 0 (*n* = 4), recipient 1 (*n* = 4), and for recipient 7 (*n* = 4) in unsuccessful treatments. *P*-value (*P <0.05) indicates statistical significance either successful or unsuccessful treatments by one-way ANOVA followed by tukey multiple comparison test. *P*-values (^†††^*P* <0.001, ^††^*P* <0.01, ^†^*P* <0.05) indicate statistical significance by Student’s *t*-test. **Fig. S5.** Microbial composition based on genus level. Relative abundances are shown as mean ± SEM for donor (*n* = 13), recipient 0 (*n* = 13), recipient 1 (*n* = 13), and recipient 7 (*n* = 13) in successful treatments, and donor (*n* = 4), recipient 0 (*n* = 4), recipient 1 (*n* = 4), and for recipient 7 (*n* = 4) in unsuccessful treatments. *P*-values (**P <0.01, *P <0.05) indicate statistical significance either successful or unsuccessful treatments by one-way ANOVA followed by tukey multiple comparison test. *P*-value (^†^*P* <0.05) indicates statistical significance by Student’s *t*-test. **Fig. S6.** Heatmap analysis based on metabolites in concentration level identified in all the groups. Each column represents a sample and each row represents a metabolite. Colors indicate fold increases or decreases in concentrations, as indicated in the key. Data are shown for donor (*n* = 9), recipient 0 (*n* = 9), recipient 1 (*n* = 9), and recipient 7 (*n* = 9) in successful treatments, and donor (*n* = 3), recipient 0 (*n* = 3), recipient 1 (*n* = 3), and for recipient 7 (*n* = 3) in unsuccessful treatments. The parameters that were used for the analysis were Euclidean distance measure and Ward cluster algorithm, using MetaboAnalyst 4.0 software. **Fig. S7.** Partial Least-Squares Discriminant Analysis (PLS-DA) parameter for the comparison of successful and unsuccessful treatments. (A) D-success vs D-failure (accuracy 0.75, R2: 0.66; Q2: 0.30); (B) R-0-success vs R-0-failure (accuracy=1, R2= 0.88; Q2= 0.73); (C) R-1-success vs R-1-failure (Accuracy 0.91; R2= 0.85; Q2= 0.58), and (D) R-7-success vs R-7-failure (Accuracy 0.75; R2= 0.8468; Q2= 0.19), shown for donor (*n* = 9), recipient 0 (*n* = 9), recipient 1 (*n* = 9), and recipient 7 (*n* = 9) in successful treatments, and donor (*n* = 3), recipient 0 (*n* = 3), recipient 1 (*n* = 3), and for recipient 7 (*n* = 3) in unsuccessful treatments. **Fig. S8.** Effect of FMT on amino acid level in successful and unsuccessful treatments. Amino acid concentration obtained from fecal sample in each group on before and after FMT. Data are shown as mean ± SEM for donor (*n* = 9), recipient 0 (*n* = 9), recipient 1 (*n* = 9), and recipient 7 (*n* = 9) in successful treatments, and donor (*n* = 3), recipient 0 (*n* = 3), recipient 1 (*n* = 3), and for recipient 7 (*n* = 3) in unsuccessful treatments and were analyzed using the unpaired t test with Welch’s correction. *P <0.05; **P <0.01. **Fig. S9.** Effect of FMT on selected metabolites in successful and unsuccessful treatments obtained from fecal sample in each group on before and after FMT. Data are shown as mean ± SEM for donor (*n* = 9), recipient 0 (*n* = 9), recipient 1 (*n* = 9), and recipient 7 (*n* = 9) in successful treatments, and donor (*n* = 3), recipient 0 (*n* = 3), recipient 1 (*n* = 3), and for recipient 7 (*n* = 3) in unsuccessful and were analyzed using the unpaired t test with Welch’s correction. *P < 0.05. GABA, gamma-Aminobutyric acid. **Fig. S10.** Abundant metabolites in lipid and fatty acid metabolism based on their relative area obtained from fecal sample in each group on before and after FMT. Data are shown as for donor (*n* = 9), recipient 0 (*n* = 9), recipient 1 (*n* = 9), and recipient 7 (*n* = 9) in successful treatments, and donor (*n* = 3), recipient 0 (*n* = 3), recipient 1 (*n* = 3), and for recipient 7 (*n* = 3) in unsuccessful treatments. Data are shown as mean ± SEM and were analyzed using the unpaired t test with Welch’s correction. *P < 0.05. **Fig. S11.** Effect of FMT on polyamines. Polyamines (spermidine and putresine) and diamine (cadaverine) in successful and unsuccessful treatments based on their concentration are obtained from fecal sample in each group on before and after FMT, for donor (*n* = 9), recipient 0 (*n* = 9), recipient 1 (*n* = 9), and recipient 7 (*n* = 9) in succeed treatments, and donor (*n* = 3), recipient 0 (*n* = 3), recipient 1 (*n* = 3), and for recipient 7 (*n* = 3) in unsuccessful treatments. Data are shown as mean ± SD. were analyzed using the unpaired t test with Welch’s correction. *P < 0.05. **Fig.S12.** Microbiota-metabolome correlation based on group specific procrustes analysis. Procrustes analysis in A) D-success group (M2 = 0.2289, *r* = 0.8781, *P* = 0.0037), B) R-0-success (M2 = 0.59, *r* = 0.6403, *P* = 0.051), C) R-1-success (M2 = 0.7202, *r* = 0.529, *P* = 0.2097), D) R-7-success (M2 = 0.2155, *r* = 0.8857, *P* = 0.0022), E) D-failure (M2 = 0.3037, *r* = 0.8345, *P* = 0.66667), F) R-0-failure (M2 = 0.1437, *r* = 0.9254, *P*= 0.5), G) R-1-failure (M2 = 0.1233, *r* = 0.9363, *P*= 0.1667), H) R-7-failure (M2 = 0.0663, *r* = 0.9661, *P*= 0.1667). **Fig. S13.** The weighted gene co-expression network analysis (WGCNA) to identify microbiota-metabolites correlation. (A) Module trait relationship among microbiota, clinical data, metabolites and fecal immunoglobulins (IgA, IgG, and IgM). Each row corresponds to a module. Each column corresponds to a trait labeled below. Each cell at the row-column intersection contains the correlation coefficient and *P*-value (in brackets) between the module and that trait. A highly positive correlation between a specific module and a trait is indicated by dark red, and green indicates negative correlation. Data are shown for donor (n = 9), recipient 0 (n = 9), recipient 1 (n = 9), and recipient 7 (n = 9) in successful treatments, and for donor (n = 3), recipient 0 (n = 3), recipient 1 (n = 3), and recipient 7 (n = 3) in unsuccessful treatments. (B) Heatmap analysis from WGCNA analysis based on modules-trait relationships. Each column represents a sample and each row represents a microbial taxon from the selected modules. Colors indicate increases or decreases in abundances, as indicated in the color key (donor and recipient n numbers in successful and unsuccessful treatments as shown above). **Fig. S14.** Heatmap based Identification of the potential microbial taxa by module trait relationship in donor cases in WGCNA analysis. Each column represents a sample and each row represents a microbial taxon from the selected modules. Colors indicate increases or decreases in abundances, as indicated in the color key, for D-success (*n* = 9) in successful treatments and D-failure (*n* = 3) in unsuccessful treatments, respectively. **Fig. S15.** The weighted gene co-expression network analysis (WGCNA) to identify microbiota-metabolites correlation before FMT. (A) Module trait relationship among microbiota, and selected metabolites. Each row corresponds to a module. Each column corresponds to a trait labeled below. Each cell at the row-column intersection contains the correlation coefficient and *P*-values (in brackets) between the module and that trait. A highly positive correlation between a specific module and a trait is indicated by dark red, and green indicates negative correlation. (B) Heatmap analysis from WGCNA analysis based on modules-trait relationships. Each column represents a sample and each row represents a microbial taxon from the selected modules. Colors indicate increases or decreases in abundances, as indicated in the color key, shown for recipient 0 (*n* = 9) in successful treatments, and recipient 0 (*n* = 3) in unsuccessful treatments, respectively. **Fig. S16.** The weighted gene co-expression network analysis (WGCNA) to identify microbiota-metabolites correlation after FMT at day 1. (A) Module trait relationship among microbiota, and selected metabolites. Each row corresponds to a module. Each column corresponds to a trait labeled below. Each cell at the row-column intersection contains the correlation coefficient and *P*-value (in brackets) between the module and that trait. A highly positive correlation between a specific module and a trait is indicated by dark red, and green indicates negative correlation. (B) Heatmap analysis from WGCNA analysis based on modules-trait relationships. Each column represents a sample and each row represents a microbial taxon from the selected modules. Colors indicate increases or decreases in abundances, as indicated in the color key, shown for recipient 1 (*n* = 9) in successful treatments, and recipient 1 (*n* = 3) in unsuccessful treatments, respectively. **Fig. S17.** Heatmap based Identification of the potential microbial taxa by module trait relationship after FMT in recipient cases in WGCNA analysis. Each column represents a sample and each row represents a microbial taxon from the selected modules. Colors indicate increases or decreases in abundances, as indicated in the color key, for recipient 7 (*n* = 9) in successful treatments, and donor (*n* = 3), recipient 7 (*n* = 3) in unsuccessful treatments, respectively. **Fig. S18.** Metagenomics analysis for potential biomarker identification for a successful FMT. (A) Diarrheal score comparison between diarrheal group for diarrhea (*n* = 49), R-0-success (*n* = 14), and R-0-failure (*n* = 6). Data are expressed as mean±SEM and were analyzed using the 1-way ANOVA with Tukey’s post hoc multiple comparisons (*P <0.05). (B) Microbial composition of overall healthy and diarrheal group for healthy (*n* = 105), diarrhea (*n* = 46), D-success (*n* = 13), D-failure (*n* = 4), R-0-success (*n* = 13), and R-0-failure (*n* = 4). Relative abundance (%) of (C) *Sporobacter*, (D) *Camphylobactor*. Data are expressed as mean ± SEM for healthy (*n* = 105), diarrhea (*n* = 46), D-success (*n* = 13), D-failure (*n* = 4), R-0-success (*n* = 13), and R-0-failure (*n* = 4).

## Data Availability

All data generated or analyzed during this study are included in this published article [and its supplementary information files]. However, further data will be made available on request to the corresponding author.

## Abbreviations

APC: Antigen-presenting cells
CD: Calf diarrhea
ELISA: Enzyme-linked immunosorbent assay
FMT: Fecal microbiota transplantation
GGT: Gamma-glutamyl transferase
KEGG: Kyoto Encyclopedia of Genes and Genomes
LDA: Linear discriminant analysis
OTU: Operational taxonomic units
PCA: Principal component analysis
PCoA: Principal coordinate analysis
RF: Random forest
VIP: Variable importance in projection
