# Supplementary material for: Development of a rational framework for the therapeutic efficacy of fecal microbiota transplantation for calf diarrhea treatment
Source: Microbiome. 2022 Feb 21;10:31. doi: 10.1186/s40168-021-01217-4 (PMC8858662; doi:10.1186/s40168-021-01217-4)
Supplement: Supplementary file 2 — Additional file 1: Table S1. Demographic information of the calves selected for FMT. Table S2. Unweighted distance matrix among groups. Table S3. P values of the Tukey’s multiple comparison test from the one-way ANOVA between PC1 scores of the PCA. Table S4. Demographic information of the selected healthy and diarrheal calves. Table S5. Unweighted unifrac distance matrix analysis. [file 40168_2021_1217_MOESM2_ESM.docx]

**Additional File 1**

**Supplementary Information for**

Development of a rational framework for understanding the efficacy

of fecal microbiota transplantation in calf diarrhea prevention

Jahidul Islam^1^, Masae Tanimizu^2^, Yu Shimizu^2^, Yoshiaki Goto^3^, Natsuki Ohtani^3^,

Kentaro Sugiyama^3^, Eriko Tatezaki^3^, Masumi Sato^4^, Eiji Makino^4^, Toru Shimada^5^,

Chise Ueda^5^, Ayumi Matsuo^1^, Yoshihisa Suyama^1^, Yoshifumi Sakai^1^, Mutsumi Furukawa^1^,

Katsuki Usami^1^, Hiroshi Yoneyama^1^, Hisashi Aso^1^, Hidekazu Tanaka^3^*, Tomonori Nochi^1,6^*

Correspondence:

Hidekazu Tanaka, D.V.M.

North Veterinary Clinical Center

Chiba Prefectural Federation of Agricultural Mutual Aid Association

99-1 Nira, Katori, Chiba 289-0407, Japan

Tel: +81-478-78-5560

Fax: +81-478-78-5565

Email: fwgc5394hide@gmail.com

Tomonori Nochi, Ph.D.

International Education and Research Center for Food and Agricultural Immunology

Graduate School of Agricultural Science, Tohoku University

468-1 Aoba, Aramaki, Aoba-ku, Sendai, Miyagi 980-8572, Japan

Tel: +81-22-757-4312

Fax: +81-22-757-4315

Email: [nochi@tohoku.ac.jp](mailto:nochi@tohoku.ac.jp)

**This PDF file includes:**

Tables S1 to S5

**Table S1. Demographic information of the calves selected for FMT.**

|  |  | Donor | | | | Recipient | | | |
| --- | --- | --- | --- | --- | --- | --- | --- | --- | --- |
|  |  | Successful (n=14) | Unsuccessful (n=6) | κ^2^ | p-value | Successful (n=14) | Unsuccessful (n=6) | κ^2^ | p-value |
| Age | Mean ±SD | 45.43 ± 37.55 | 597.17 ± 1091.77 | - | *ns* | 18.85 ± 12.41 | 112± 81.77 | - | <0.05 |
| Strain | Holstein | 5 | 2 | 0.042 | *ns* | 5 | 1 | 0.7937 | *ns* |
|  | F1 | 6 | 3 |  |  | 3 | 2 |  |  |
|  | Wagyu | 3 | 1 |  |  | 6 | 3 |  |  |
| Sex | Male | 5 | 2 | 0.010 | *ns* | 7 | 6 | 4.047 | <0.05 |
|  | Female | 9 | 4 |  |  | 7 | 0 |  |  |

*ns*: not significant

**Table S2.** **Unweighted distance matrix among groups.**

| Group 1 | Group 2 | pseudo-F | p-value | q-value |
| --- | --- | --- | --- | --- |
| D0-failure | D0-success | 2.825119 | 0.003 | 0.006 |
| D0-failure | R0-failure | 0.721152 | 0.476 | 0.605818 |
| D0-failure | R0-success | 4.401998 | 0.002 | 0.004308 |
| D0-failure | R1-failure | 0.708105 | 0.758 | 0.884333 |
| D0-failure | R1-success | 5.397835 | 0.002 | 0.004308 |
| D0-failure | R7-failure | 0.732953 | 0.71 | 0.864348 |
| D0-failure | R7-success | 3.598423 | 0.001 | 0.004308 |
| D0-success | R0-failure | 2.171651 | 0.012 | 0.017684 |
| D0-success | R0-success | 4.504881 | 0.001 | 0.004308 |
| D0-success | R1-failure | 2.438763 | 0.005 | 0.009333 |
| D0-success | R1-success | 4.968165 | 0.002 | 0.004308 |
| D0-success | R7-failure | 2.305558 | 0.009 | 0.01575 |
| D0-success | R7-success | 1.200246 | 0.244 | 0.325333 |
| R0-failure | R0-success | 3.761136 | 0.002 | 0.004308 |
| R0-failure | R1-failure | 0.396214 | 0.808 | 0.90496 |
| R0-failure | R1-success | 4.538317 | 0.002 | 0.004308 |
| R0-failure | R7-failure | 0.462307 | 0.945 | 0.961 |
| R0-failure | R7-success | 2.713068 | 0.011 | 0.017111 |
| R0-success | R1-failure | 4.61005 | 0.001 | 0.004308 |
| R0-success | R1-success | 0.540048 | 0.961 | 0.961 |
| R0-success | R7-failure | 4.224889 | 0.002 | 0.004308 |
| R0-success | R7-success | 2.167637 | 0.015 | 0.021 |
| R1-failure | R1-success | 5.672492 | 0.001 | 0.004308 |
| R1-failure | R7-failure | 0.485155 | 0.938 | 0.961 |
| R1-failure | R7-success | 3.13735 | 0.001 | 0.004308 |
| R1-success | R7-failure | 5.289244 | 0.001 | 0.004308 |
| R1-success | R7-success | 2.312445 | 0.01 | 0.016471 |
| R7-failure | R7-success | 2.966907 | 0.002 | 0.004308 |

Table S3. P values of the Tukey’s multiple comparison test from the one-way ANOVA between PC1 scores of the PCA

| Tukey's Multiple Comparison Test | Mean Diff. | q | Summary |
| --- | --- | --- | --- |
| D-failure vs D-success | -9.16 | 3.342 | ns |
| D-failure vs R-0-failure | -0.08107 | 0.02415 | ns |
| D-failure vs R-0-success | -18.47 | 6.738 | *** |
| D-failure vs R-1-failure | -3.364 | 1.002 | ns |
| D-failure vs R-1-success | -15.31 | 5.586 | ** |
| D-failure vs R-7-failure | -4.704 | 1.401 | ns |
| D-failure vs R-7-success | -12.33 | 4.498 | ns |
| D-success vs R-0-failure | 9.079 | 3.313 | ns |
| D-success vs R-0-success | -9.307 | 4.803 | * |
| D-success vs R-1-failure | 5.796 | 2.115 | ns |
| D-success vs R-1-success | -6.15 | 3.173 | ns |
| D-success vs R-7-failure | 4.456 | 1.626 | ns |
| D-success vs R-7-success | -3.168 | 1.635 | ns |
| R-0-failure vs R-0-success | -18.39 | 6.709 | *** |
| R-0-failure vs R-1-failure | -3.283 | 0.978 | ns |
| R-0-failure vs R-1-success | -15.23 | 5.556 | ** |
| R-0-failure vs R-7-failure | -4.623 | 1.377 | ns |
| R-0-failure vs R-7-success | -12.25 | 4.468 | ns |
| R-0-success vs R-1-failure | 15.1 | 5.511 | ** |
| R-0-success vs R-1-success | 3.157 | 1.629 | ns |
| R-0-success vs R-7-failure | 13.76 | 5.022 | * |
| R-0-success vs R-7-success | 6.139 | 3.168 | ns |
| R-1-failure vs R-1-success | -11.95 | 4.359 | ns |
| R-1-failure vs R-7-failure | -1.34 | 0.3992 | ns |
| R-1-failure vs R-7-success | -8.964 | 3.271 | ns |
| R-1-success vs R-7-failure | 10.61 | 3.87 | ns |
| R-1-success vs R-7-success | 2.982 | 1.539 | ns |
| R-7-failure vs R-7-success | -7.624 | 2.782 | ns |

**Table S4.** **Demographic information of the selected healthy and diarrheal calves**

|  |  | Healthy (n=109) | Diarrheal (n=49) | κ^2^ | P-value |
| --- | --- | --- | --- | --- | --- |
| Age (days) | Mean ±SD | 30.55 ± 19.20 | 21.86 ± 14.02 | - | <0.05 |
| Strain | Holstein | 53 | 10 | 11.5 | <0.01 |
|  | F1 | 33 | 21 |  |  |
|  | Wagyu | 23 | 18 |  |  |
| Sex | Male | 35 | 25 | 5.132 | <0.05 |
|  | Female | 74 | 24 |  |  |

**Table S5.** **Unweighted unifrac distance matrix analysis**

| Group 1 | Group 2 | pseudo-F | p-value | q-value |
| --- | --- | --- | --- | --- |
| Diarrhea | R-0-failure | 3.513249973 | 0.004 | 0.0075 |
| Diarrhea | R-0-success | 0.850325082 | 0.622 | 0.622 |
| Diarrhea | Healthy | 4.639829147 | 0.001 | 0.005 |
| Diarrhea | D-failure | 4.435816093 | 0.002 | 0.006 |
| Diarrhea | D-success | 3.594316099 | 0.001 | 0.005 |
| R-0-failure | R-0-success | 4.275042193 | 0.006 | 0.009 |
| R-0-failure | Healthy | 2.545770535 | 0.01 | 0.013636364 |
| R-0-failure | D-failure | 0.726819104 | 0.488 | 0.522857143 |
| R-0-failure | D-success | 2.264926021 | 0.013 | 0.01625 |
| R-0-success | Healthy | 3.756782285 | 0.002 | 0.006 |
| R-0-success | D-failure | 4.935567692 | 0.003 | 0.0075 |
| R-0-success | D-success | 4.150051069 | 0.001 | 0.005 |
| Healthy | D-failure | 3.17415265 | 0.004 | 0.0075 |
| Healthy | D-success | 1.307085075 | 0.157 | 0.181153846 |
| D-failure | D-success | 2.919250448 | 0.006 | 0.009 |
