# Supplementary material for: Development of a rational framework for the therapeutic efficacy of fecal microbiota transplantation for calf diarrhea treatment
Source: Microbiome. 2022 Feb 21;10:31. doi: 10.1186/s40168-021-01217-4 (PMC8858662; doi:10.1186/s40168-021-01217-4)
Supplement: Supplementary file 5 — Additional file 4: Fig. S1. Overview of farm specific cow and disease condition. The effect of FMT on enteritis and weak calf syndrome disease prevention in successful and unsuccessful treatments. (A) Farm specific cows’ number. (B) Success rate evaluated by presence and absence of enteritis and weak calf syndrome before and after FMT for recipient (n = 14) in successful treatments and recipient (n = 6) in unsuccessful treatments. ‘+, presence; ‘-, absence. Fig. S2. Clinical parameter. Clinical efficacy of FMT on successful and unsuccessful treatments based on day-0 (before FMT) and day-7 (after FMT). Data are expressed as mean±SD, shown for R-0-success (n = 14), R-0-failure (n = 6), R-7-success (n = 14), and R-7-failure (n = 4). γ-GT, Gamma-Glutamyl Transferase; MCV, mean corpuscular volume; MCH, mean corpuscular hemoglobin; MCHC, mean corpuscular hemoglobin concentration. *P <0.05 (Student’s t-test). Fig. S3. Microbial composition based on phylum level. Relative abundances are shown as mean ± SEM for donor (n = 13), recipient 0 (n = 13), recipient 1 (n = 13), and recipient 7 (n = 13) in successful treatments, and donor (n = 4), recipient 0 (n = 4), recipient 1 (n = 4), and for recipient 7 (n = 4) in unsuccessful treatments. P-values (*P <0.05, **P <0.01) indicate statistical significance either successful or unsuccessful treatments by one-way ANOVA followed by tukey multiple comparison test. P-values (††P <0.01, †P <0.05) indicate statistical significance by Student’s t-test. Fig. S4. Microbial composition based on family level. Relative abundances are shown as mean ± SEM for donor (n = 13), recipient 0 (n = 13), recipient 1 (n = 13), and recipient 7 (n = 13) in successful treatments, and donor (n = 4), recipient 0 (n = 4), recipient 1 (n = 4), and for recipient 7 (n = 4) in unsuccessful treatments. P-value (*P <0.05) indicates statistical significance either successful or unsuccessful treatments by one-way ANOVA followed by tukey multiple comparison test. P-value [file 40168_2021_1217_MOESM5_ESM.pdf]

Successful

No	Dietary enteritis (0 days after FMT)	Weak calf syndrome (0 days after FMT)	Dietary enteritis (7 days after FMT)	Weak calf syndrome (7 days after FMT)
1	+	-	-	-
2	-	-	-	-
3	-	-	-	-
4	-	-	-	-
5	+	-	-	-
6	-	-	-	-
7	-	-	-	-
8	-	-	-	-
9	+	-	-	-
10	-	-	-	-
11	-	-	-	-
12	-	-	-	-
13	-	-	-	-
14	+	-	-	-

Unsuccessful

No	Dietary enteritis (0 days after FMT)	Weak calf syndrome (0 days after FMT)	Dietary enteritis (7 days after FMT)	Weak calf syndrome (7 days after FMT)
15	+	-	+	-
16	-	-	-	-
17	-	-	-	-
18	-	+	-	+
19	-	-	X	X
20	-	-	X	X

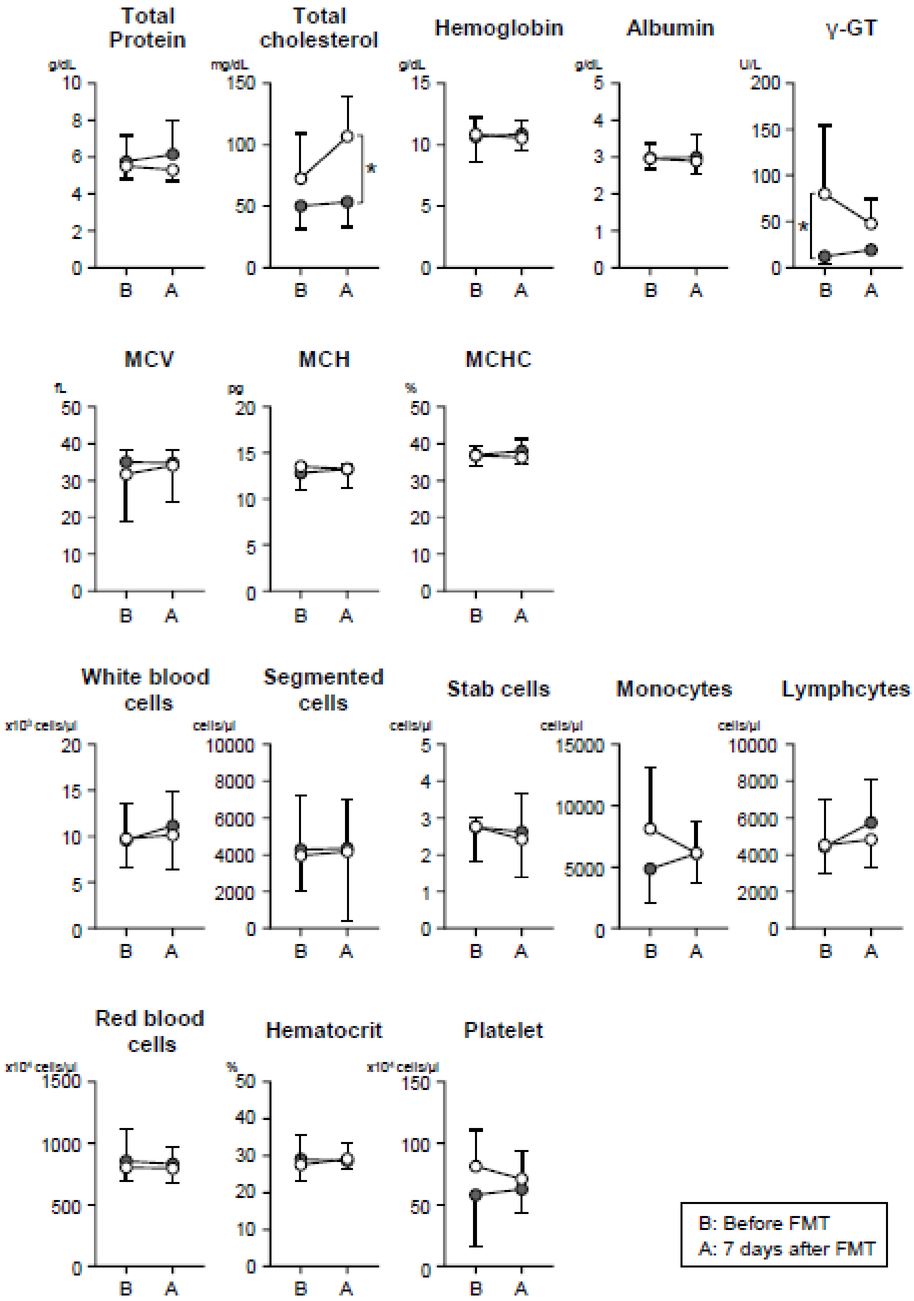

Total Protein
Total cholesterol
Hemoglobin
Albumin
γ-GT
MCV
MCH
MCHC
White blood cells
Segmented cells
Stab cells
Monocytes
Lymphcytes
Red blood cells
Hematocrit
Platelet
B: Before FMT
A: 7 days after FMT

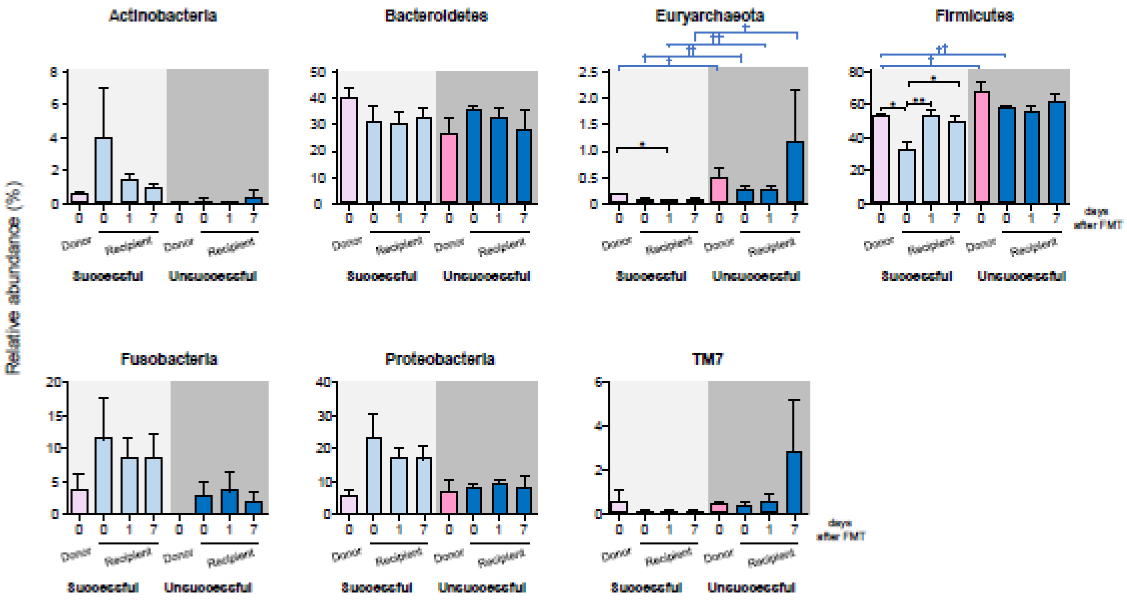
Actinobacteria
Bacteroidetes
Euryarchaeota
Firmicutes
Fusobacteria
Proteobacteria
TM7
Relative abundance (%)
Donor
Recipient
Successful
Unsuccessful
days after FMT

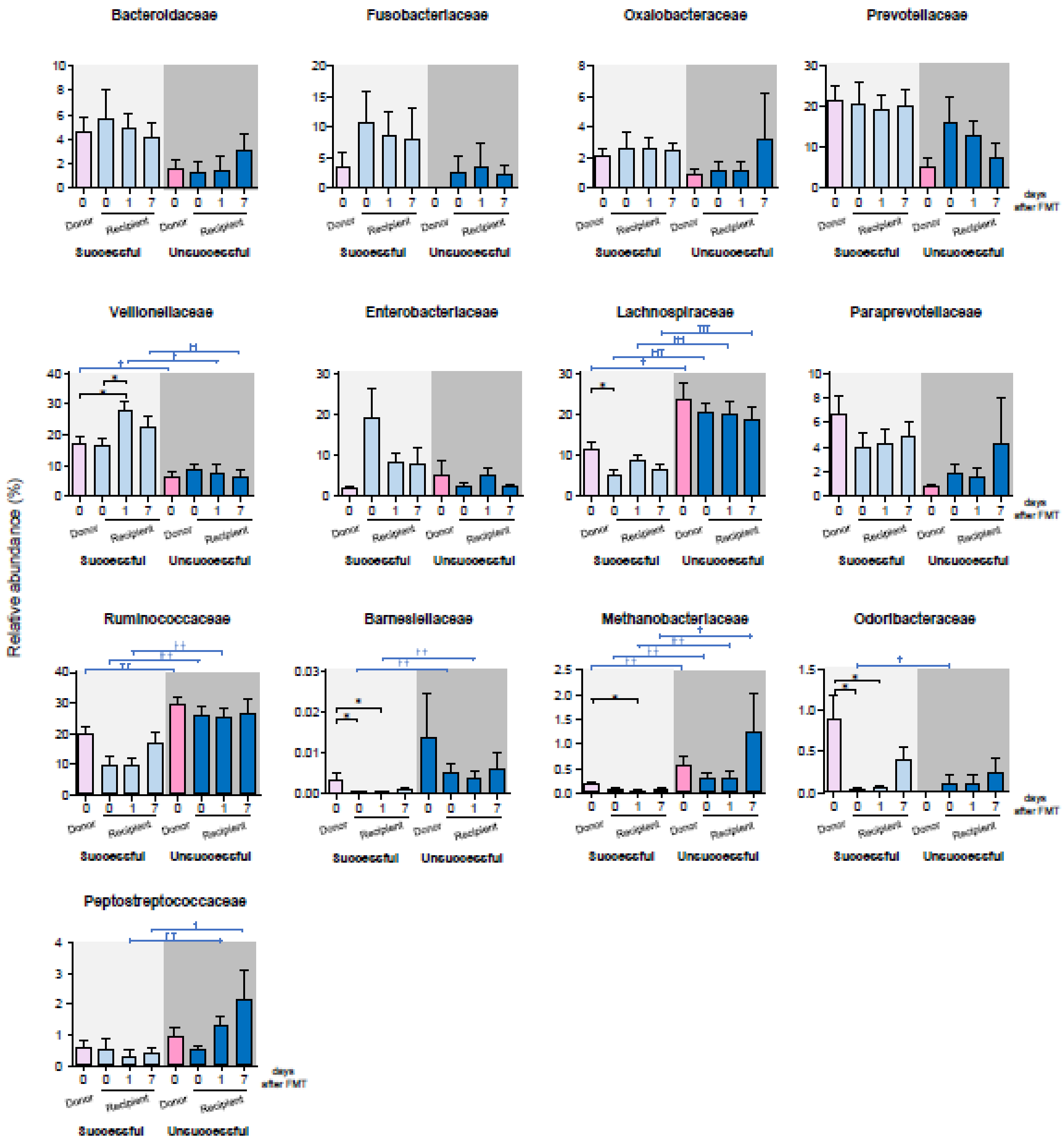

Bacteroidaceae
Fusobacteriaceae
Oxalobacteraceae
Prevotellaceae
Veillonellaceae
Enterobacteriaceae
Lachnospiraceae
Paraprevotellaceae
Ruminococcaceae
Barnesiellaceae
Methanobacteriaceae
Odoribacteraceae
Peptostreptococcaceae
Relative abundance (%)
Donor
Recipient
Successful
Unsuccessful
days after FMT

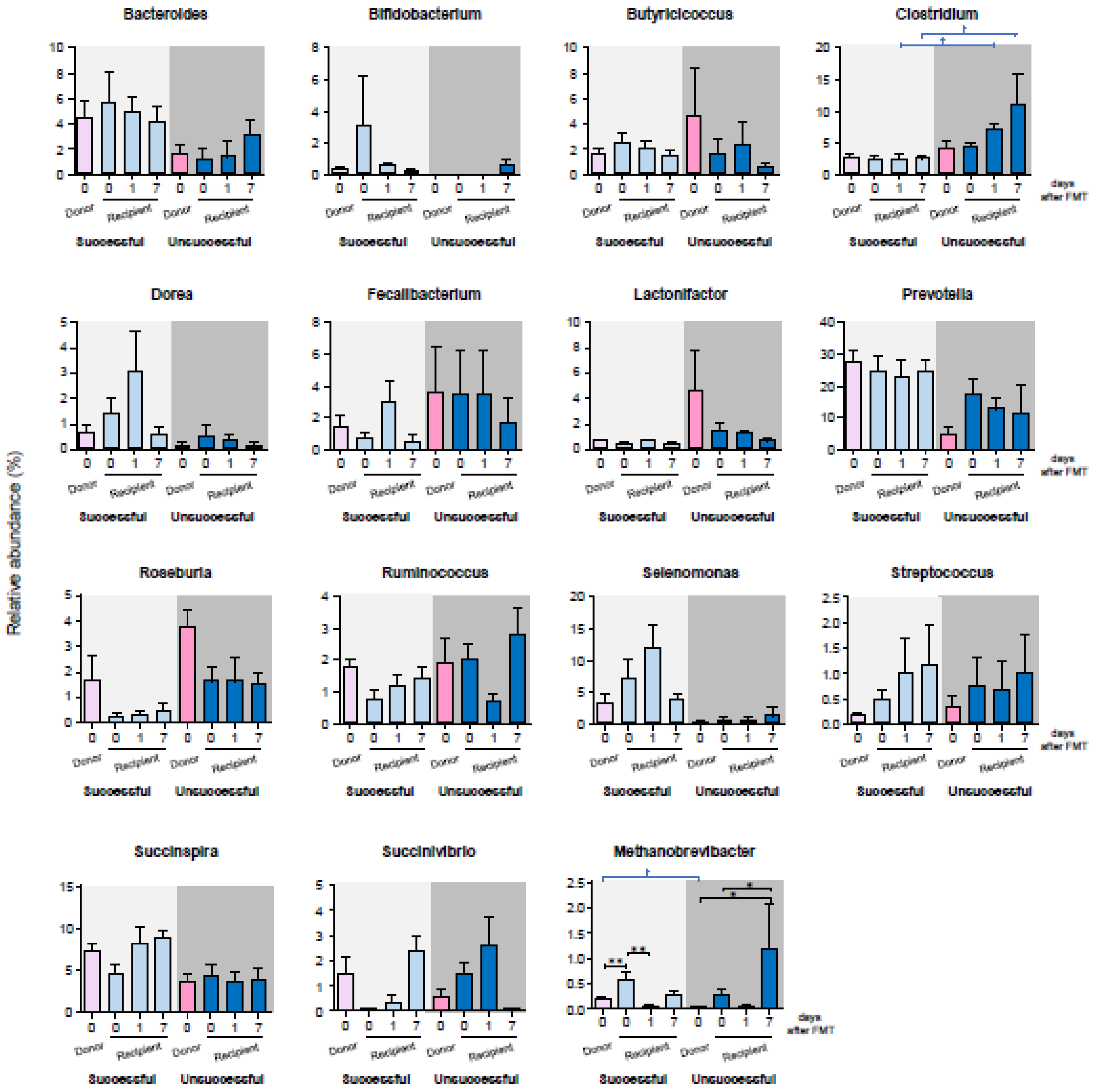

Relative abundance (%)
Bacteroides
Bifidobacterium
Butyricicoccus
Clostridium
Dorea
Fecalibacterium
Lactonifactor
Prevotella
Roseburia
Ruminococcus
Selenomonas
Streptococcus
Succinspira
Succinivibrio
Methanobrevibacter
Donor
Recipient
Successful
Unsuccessful
days after FMT

Figure S6

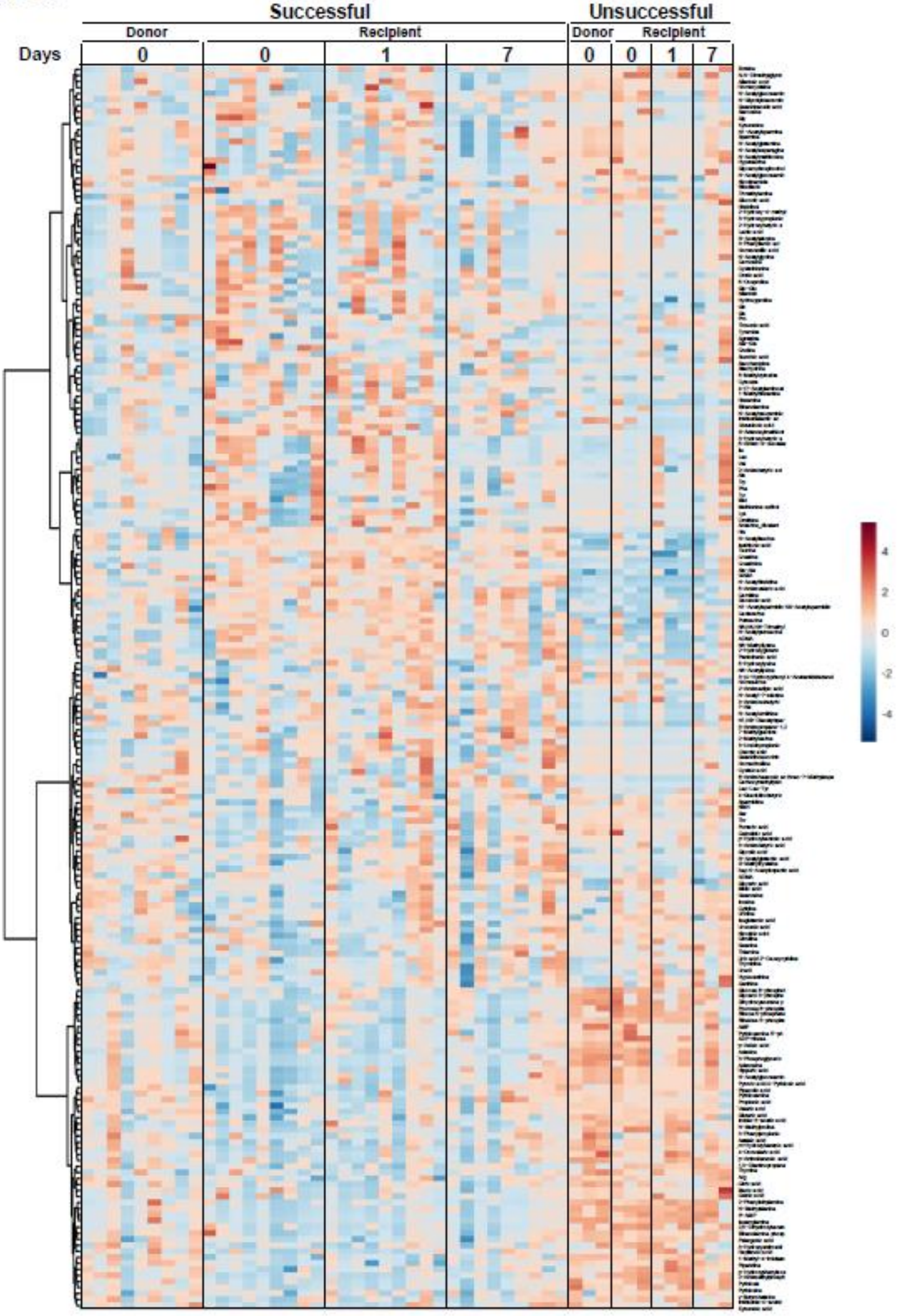

Successful
Unsuccessful
Donor
Recipient
Donor
Recipient
Days
0
0
1
7
0
0
1
7
4
2
0
-2
-4

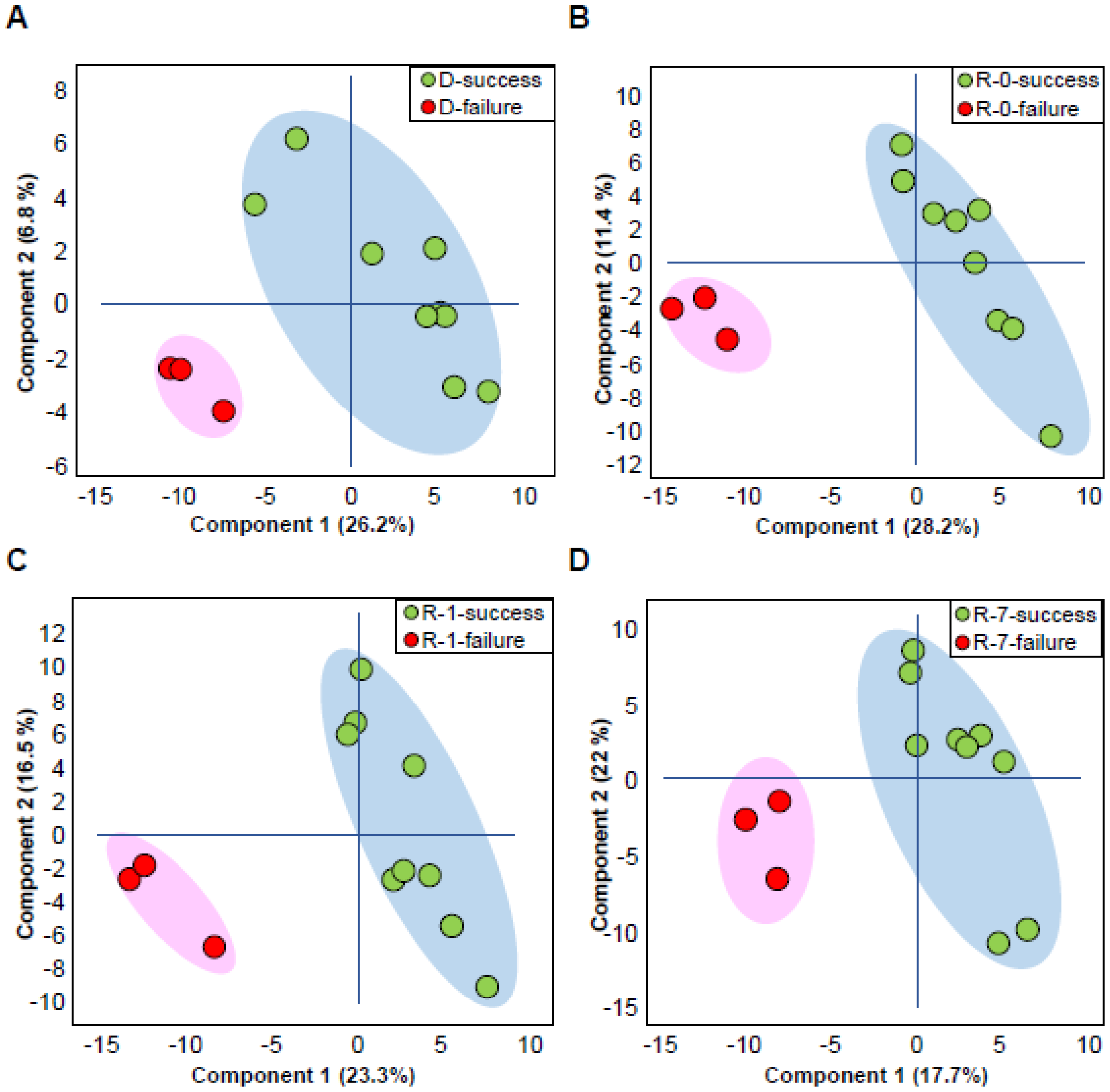

A
D-success
D-failure
Component 2 (6.8 %)
Component 1 (26.2%)
B
R-0-success
R-0-failure
Component 2 (11.4 %)
Component 1 (28.2%)
C
R-1-success
R-1-failure
Component 2 (16.5 %)
Component 1 (23.3%)
D
R-7-success
R-7-failure
Component 2 (22 %)
Component 1 (17.7%)

Figure 3[illegible]

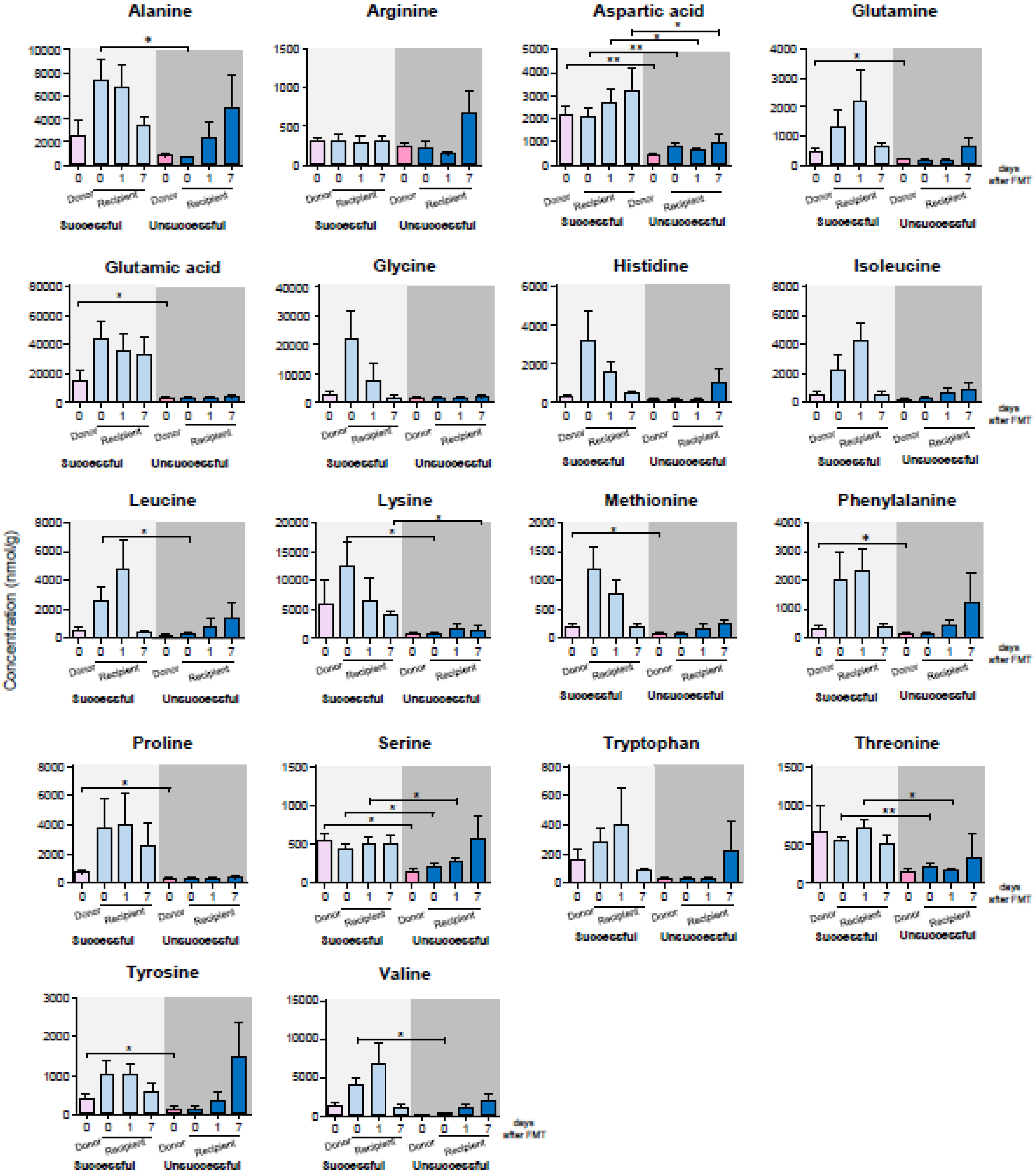
Alanine
Arginine
Aspartic acid
Glutamine
Glutamic acid
Glycine
Histidine
Isoleucine
Leucine
Lysine
Methionine
Phenylalanine
Proline
Serine
Tryptophan
Threonine
Tyrosine
Valine
Concentration (nmol/g)
0 0 1 7 0 0 1 7
Donor
Recipient
Successful
Unsuccessful
days after FMT

Figure S9

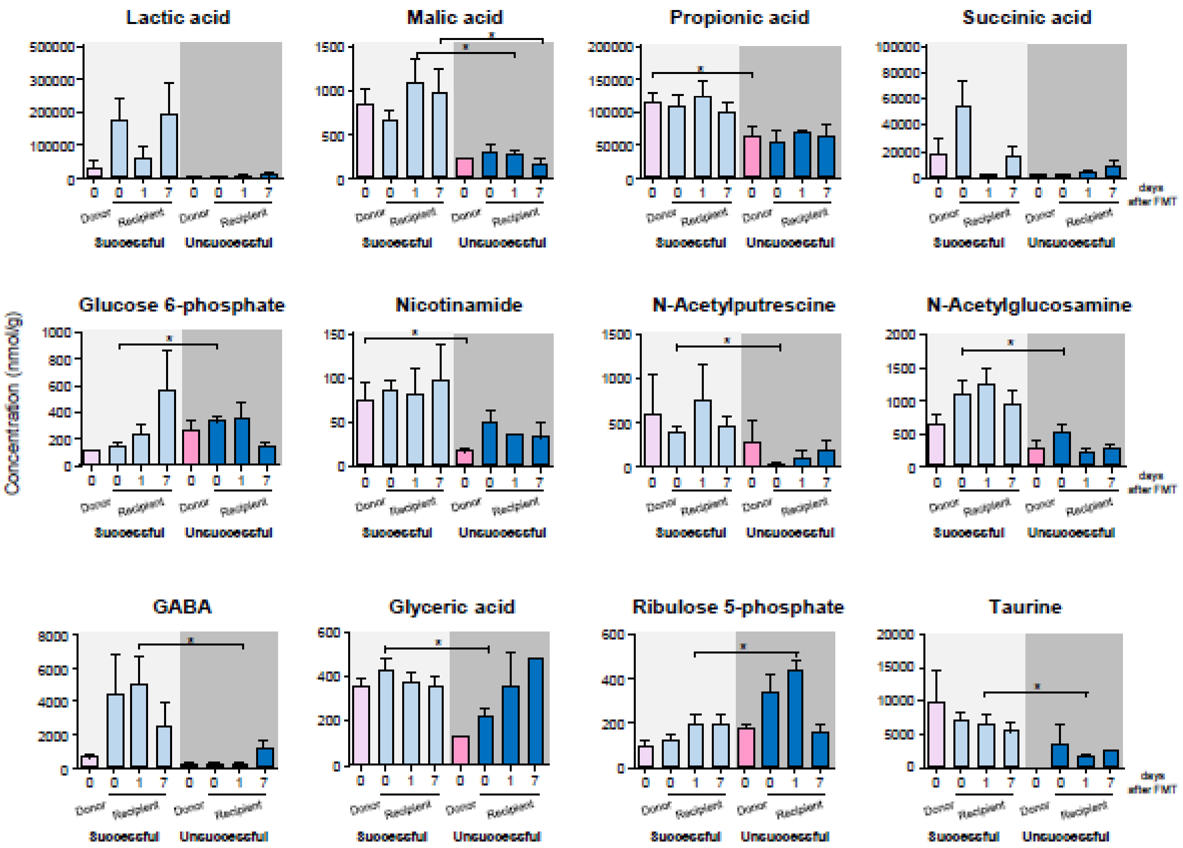
Lactic acid
Malic acid
Propionic acid
Succinic acid
Glucose 6-phosphate
Nicotinamide
N-Acetylputrescine
N-Acetylglucosamine
GABA
Glyceric acid
Ribulose 5-phosphate
Taurine
Concentration (nmol/g)
Donor
Recipient
Successful
Unsuccessful
days after FMT

Figure S10

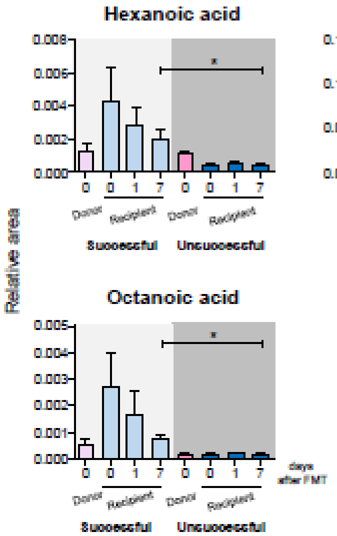

Hexanoic acid
Choline
Cholic acid
Butyric acid
Octanoic acid
Relative area
Donor
Recipient
Successful
Unsuccessful
days after FMT

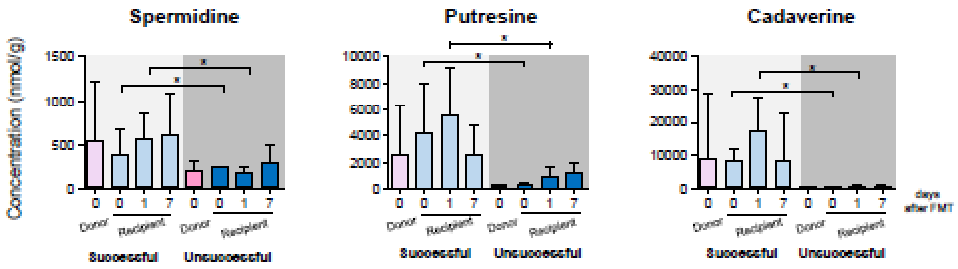

Spermidine
Putresine
Cadaverine
Concentration (nmol/g)
Donor
Recipient
Successful
Unsuccessful
days after FMT

Figure S12

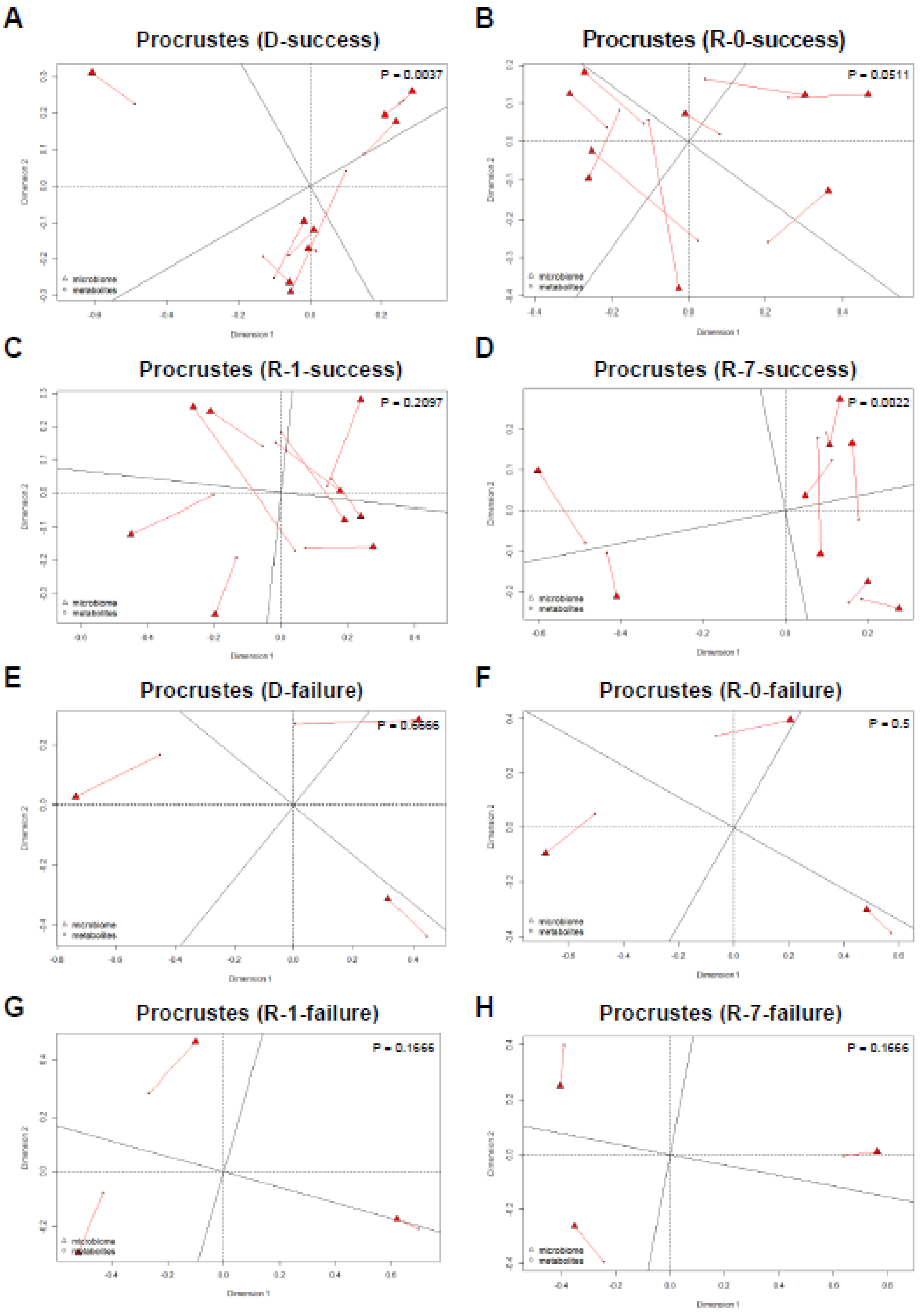

A
Procrustes (D-success)
P = 0.0037
B
Procrustes (R-0-success)
P = 0.0511
C
Procrustes (R-1-success)
P = 0.2097
D
Procrustes (R-7-success)
P = 0.0022
E
Procrustes (D-failure)
P = 0.6666
F
Procrustes (R-0-failure)
P = 0.5
G
Procrustes (R-1-failure)
P = 0.1666
H
Procrustes (R-7-failure)
P = 0.1666
microbiome
metabolites
Dimension 1
Dimension 2

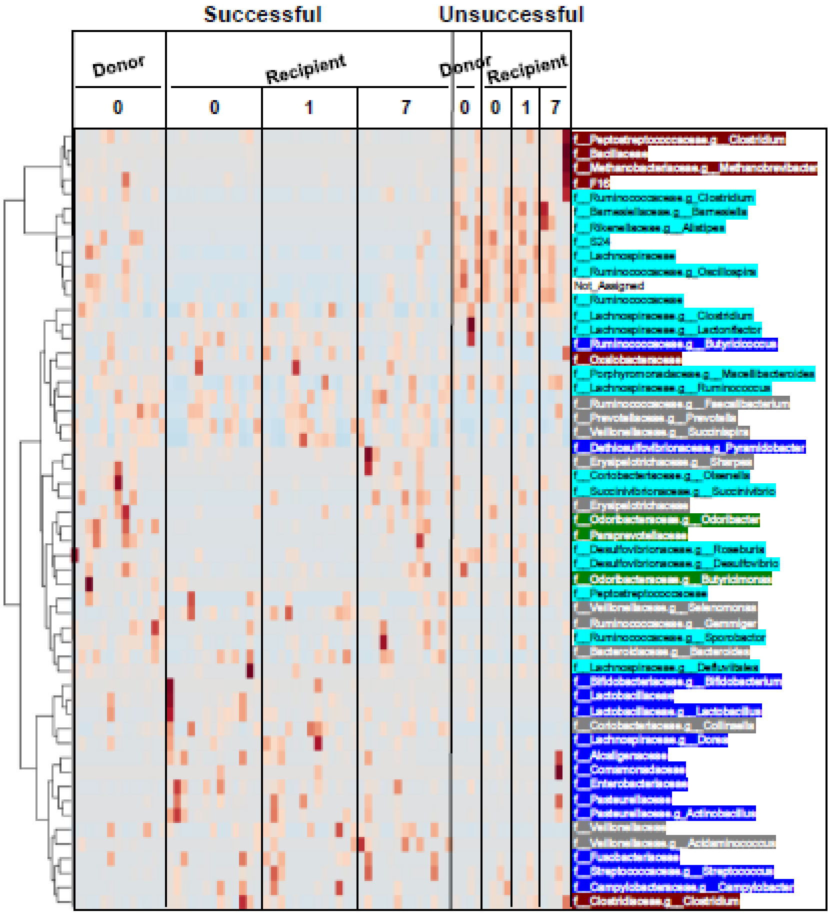
Successful
Unsuccessful
Donor
Recipient
Donor
Recipient
0
0
1
7
0
0
1
7
f__Peptostreptococcaceae.g__Clostridium
f__Bacillaceae
f__Methanobacteriaceae.g__Methanobrevibacter
f__F16
f__Ruminococcaceae.g_Clostridium
f__Barnesiellaceae.g__Barnesiella
f__Rikenellaceae.g__Alistipes
f__S24
f__Lachnospiraceae
f__Ruminococcaceae.g_Oscillospira
Not_Assigned
f__Ruminococcaceae
f__Lachnospiraceae.g__Clostridium
f__Lachnospiraceae.g__Lactonifactor
f__Ruminococcaceae.g__Butyricicoccus
f__Oxalobacteraceae
f__Porphyromonadaceae.g__Macellibacteroides
f__Lachnospiraceae.g__Ruminococcus
f__Ruminococcaceae.g__Faecalibacterium
f__Prevotellaceae.g__Prevotella
f__Veillonellaceae.g__Succinispira
f__Dethiosulfovibrionaceae.g_Pyramidobacter
f__Erysipelotrichaceae.g__Sharpea
f__Coriobacteriaceae.g__Olsenella
f__Succinivibrionaceae.g__Succinivibrio
f__Erysipelotrichaceae
f__Odoribacteraceae.g__Odoribacter
f__Paraprevotellaceae
f__Desulfovibrionaceae.g__Roseburia
f__Desulfovibrionaceae.g__Desulfovibrio
f__Odoribacteraceae.g__Butyricimonas
f__Peptostreptococcaceae
f__Veillonellaceae.g__Selenomonas
f__Ruminococcaceae.g__Gemmiger
f__Ruminococcaceae.g__Sporobacter
f__Bacteroidaceae.g__Bacteroides
f__Lachnospiraceae.g__Defluviitalea
f__Bifidobacteriaceae.g__Bifidobacterium
f__Lactobacillaceae
f__Lactobacillaceae.g__Lactobacillus
f__Coriobacteriaceae.g__Collinsella
f__Lachnospiraceae.g__Dorea
f__Alcaligenaceae
f__Comamonadaceae
f__Enterobacteriaceae
f__Pasteurellaceae
f__Pasteurellaceae.g_Actinobacillus
f__Veillonellaceae
f__Veillonellaceae.g__Acidaminococcus
f__Fusobacteriaceae
f__Streptococcaceae.g__Streptococcus
f__Campylobacteraceae.g__Campylobacter
f__Clostridiaceae.g__Clostridium

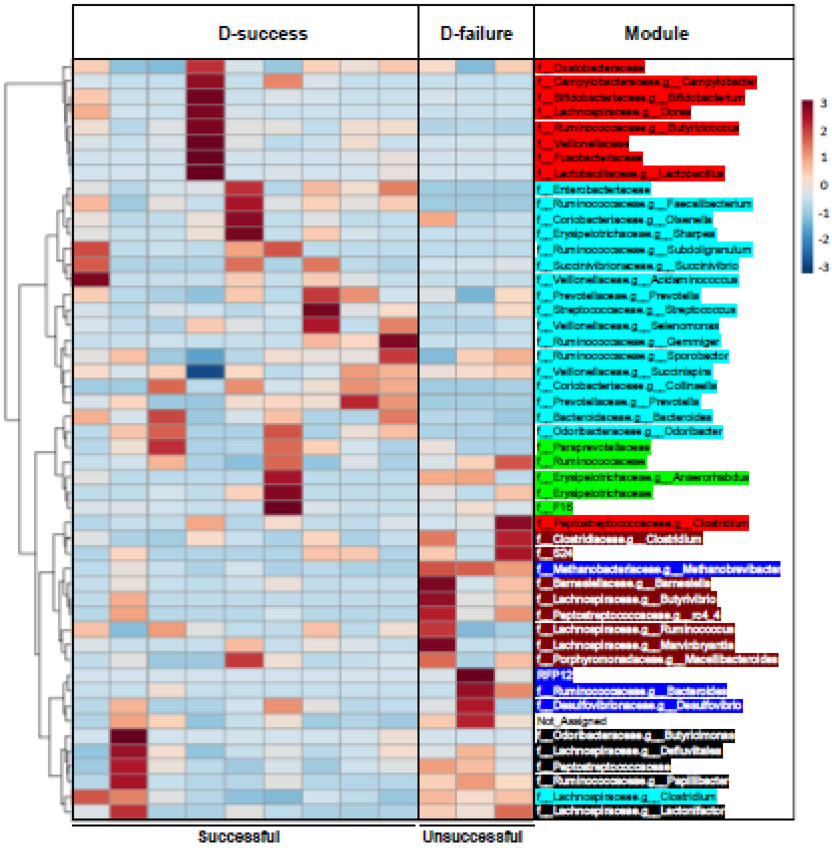

D-success
D-failure
Module
f__Oxalobacteraceae
f__Campylobacteraceae.g__Campylobacter
f__Bifidobacteriaceae.g__Bifidobacterium
f__Lachnospiraceae.g__Dorea
f__Ruminococcaceae.g__Butyricicoccus
f__Veillonellaceae
f__Fusobacteriaceae
f__Lactobacillaceae.g__Lactobacillus
f__Enterobacteriaceae
f__Ruminococcaceae.g__Faecalibacterium
f__Coriobacteriaceae.g__Olsenella
f__Erysipelotrichaceae.g__Sharpea
f__Ruminococcaceae.g__Subdoligranulum
f__Succinivibrionaceae.g__Succinivibrio
f__Veillonellaceae.g__Acidaminococcus
f__Prevotellaceae.g__Prevotella
f__Streptococcaceae.g__Streptococcus
f__Veillonellaceae.g__Selenomonas
f__Ruminococcaceae.g__Gemmiger
f__Ruminococcaceae.g__Sporobacter
f__Veillonellaceae.g__Succinispira
f__Coriobacteriaceae.g__Collinsella
f__Prevotellaceae.g__Prevotella
f__Bacteroidaceae.g__Bacteroides
f__Odoribacteraceae.g__Odoribacter
f__Paraprevotellaceae
f__Ruminococcaceae
f__Erysipelotrichaceae.g__Anaerorhabdus
f__Erysipelotrichaceae
f__F16
f__Peptostreptococcaceae.g__Clostridium
f__Clostridiaceae.g__Clostridium
f__S24
f__Methanobacteriaceae.g__Methanobrevibacter
f__Barnesiellaceae.g__Barnesiella
f__Lachnospiraceae.g__Butyrivibrio
f__Peptostreptococcaceae.g__rc4_4
f__Lachnospiraceae.g__Ruminococcus
f__Lachnospiraceae.g__Marvinbryantia
f__Porphyromonadaceae.g__Macellibacteroides
RFP12
f__Ruminococcaceae.g__Bacteroides
f__Desulfovibrionaceae.g__Desulfovibrio
Not_Assigned
f__Odoribacteraceae.g__Butyricimonas
f__Lachnospiraceae.g__Defluviitalea
f__Peptostreptococcaceae
f__Ruminococcaceae.g__Papillibacter
f__Lachnospiraceae.g__Clostridium
f__Lachnospiraceae.g__Lactonifactor
3
2
1
0
-1
-2
-3
Successful
Unsuccessful

Figure S15

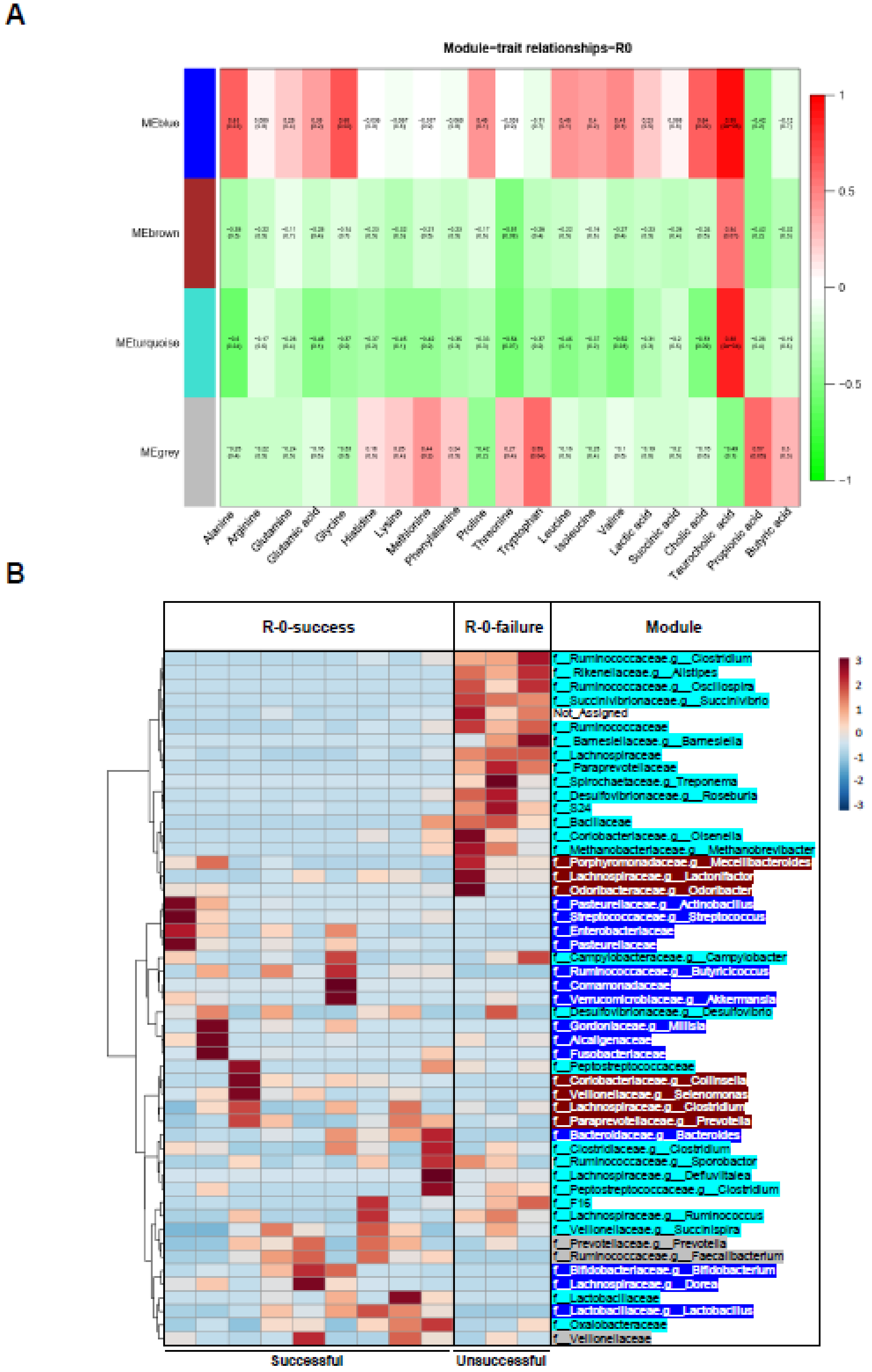
A
Module-trait relationships-R0
MEblue
MEbrown
MEturquoise
MEgrey
Alanine
Arginine
Glutamine
Glutamic acid
Glycine
Histidine
Lysine
Methionine
Phenylalanine
Proline
Threonine
Tryptophan
Leucine
Isoleucine
Valine
Lactic acid
Succinic acid
Cholic acid
Taurocholic acid
Propionic acid
Butyric acid
1
0.5
0
−0.5
−1
B
R-0-success
R-0-failure
Module
f__Ruminococcaceae.g__Clostridium
f__Rikenellaceae.g__Alistipes
f__Ruminococcaceae.g__Oscillospira
f__Succinivibrionaceae.g__Succinivibrio
Not_Assigned
f__Ruminococcaceae
f__Barnesiellaceae.g__Barnesiella
f__Lachnospiraceae
f__Paraprevotellaceae
f__Spirochaetaceae.g_Treponema
f__Desulfovibrionaceae.g__Roseburia
f__S24
f__Bacillaceae
f__Coriobacteriaceae.g__Olsenella
f__Methanobacteriaceae.g__Methanobrevibacter
f__Porphyromonadaceae.g__Mecelibacteroides
f__Lachnospiraceae.g__Lactonifactor
f__Odoribacteraceae.g__Odoribacter
f__Pasteurellaceae.g__Actinobacillus
f__Streptococcaceae.g__Streptococcus
f__Enterobacteriaceae
f__Pasteurellaceae
f__Campylobacteraceae.g__Campylobacter
f__Ruminococcaceae.g__Butyricicoccus
f__Comamonadaceae
f__Verrucomicrobiaceae.g__Akkermansia
f__Desulfovibrionaceae.g__Desulfovibrio
f__Gordoniaceae.g__Millisia
f__Alcaligenaceae
f__Fusobacteriaceae
f__Peptostreptococcaceae
f__Coriobacteriaceae.g__Collinsella
f__Veillonellaceae.g__Selenomonas
f__Lachnospiraceae.g__Clostridium
f__Paraprevotellaceae.g__Prevotella
f__Bacteroidaceae.g__Bacteroides
f__Clostridiaceae.g__Clostridium
f__Ruminococcaceae.g__Sporobacter
f__Lachnospiraceae.g__Defluviitalea
f__Peptostreptococcaceae.g__Clostridium
f__F16
f__Lachnospiraceae.g__Ruminococcus
f__Veillonellaceae.g__Succinispira
f__Prevotellaceae.g__Prevotella
f__Ruminococcaceae.g__Faecalibacterium
f__Bifidobacteriaceae.g__Bifidobacterium
f__Lachnospiraceae.g__Dorea
f__Lactobacillaceae
f__Lactobacillaceae.g__Lactobacillus
f__Oxalobacteraceae
f__Veillonellaceae
3
2
1
0
-1
-2
-3
Successful
Unsuccessful

Figure S16

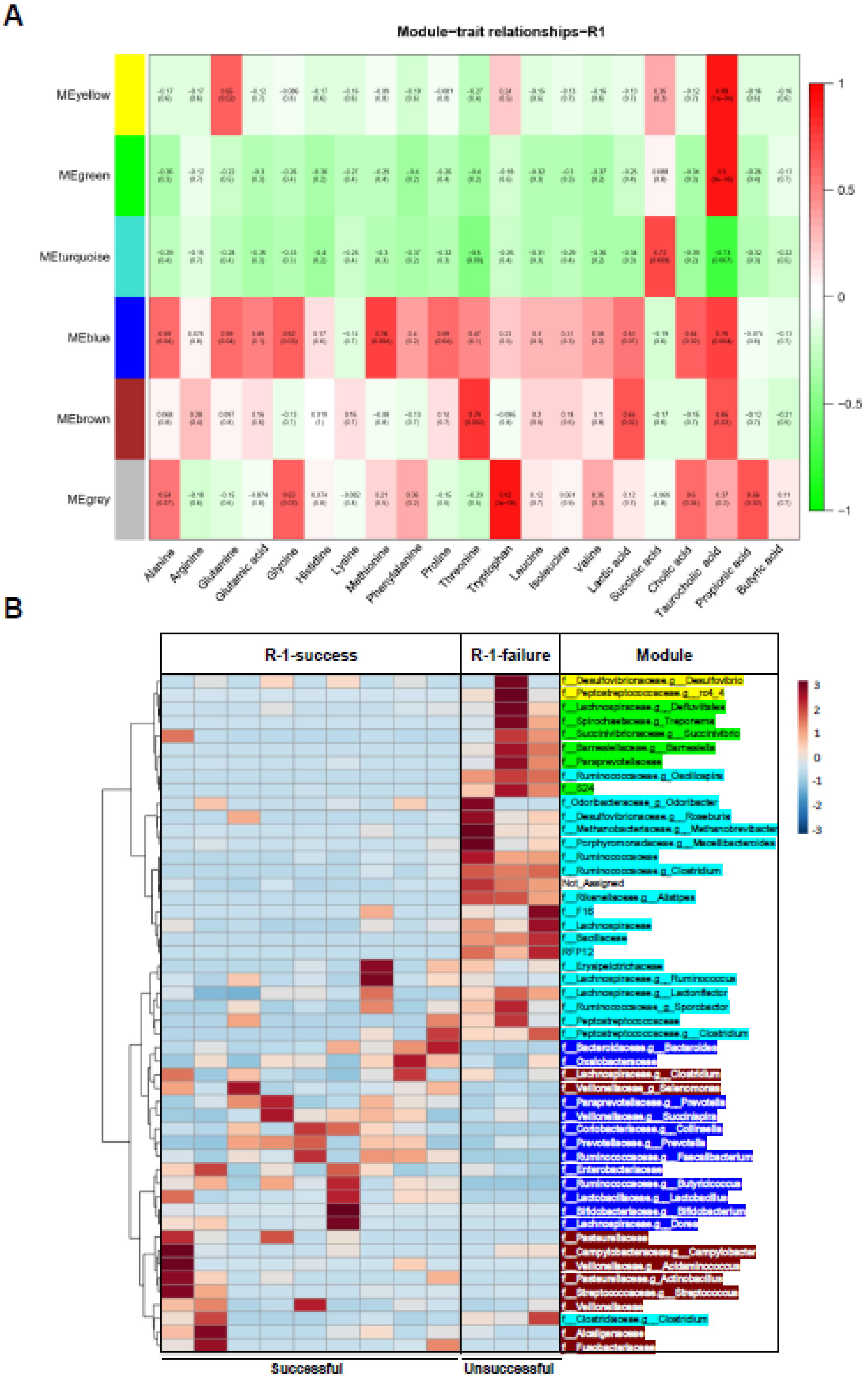
A
Module−trait relationships−R1
MEyellow
MEgreen
MEturquoise
MEblue
MEbrown
MEgrey
Alanine
Arginine
Glutamine
Glutamic acid
Glycine
Histidine
Lysine
Methionine
Phenylalanine
Proline
Threonine
Tryptophan
Leucine
Isoleucine
Valine
Lactic acid
Succinic acid
Cholic acid
Taurocholic acid
Propionic acid
Butyric acid
1
0.5
0
−0.5
−1
B
R-1-success
R-1-failure
Module
Successful
Unsuccessful
Not_Assigned
RFP12
3
2
1
0
-1
-2
-3

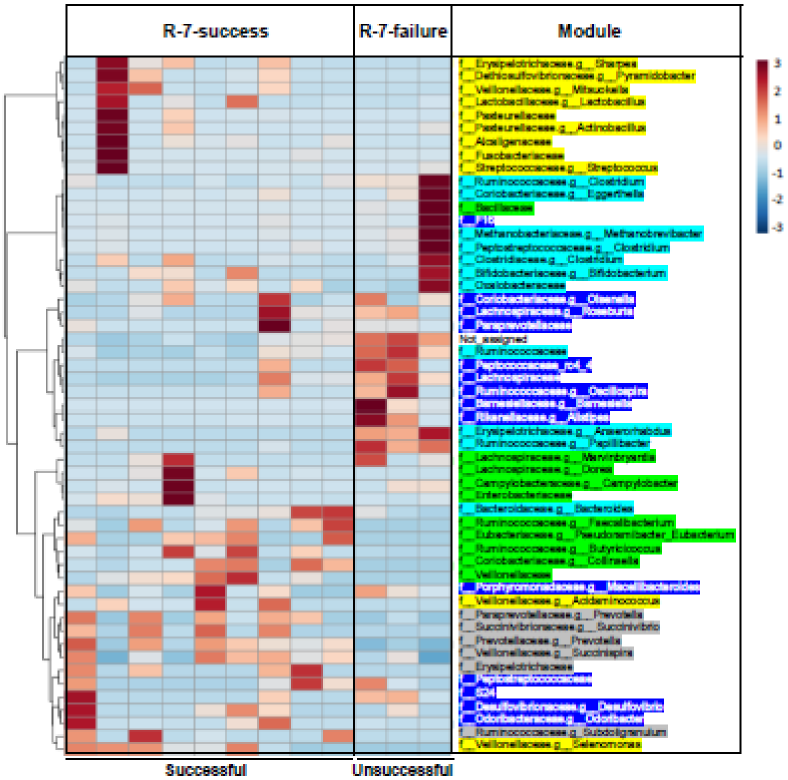

R-7-success
R-7-failure
Module
f__Erysipelotrichaceae.g__Sharpea
f__Dethiosulfovibrionaceae.g__Pyramidobacter
f__Veillonellaceae.g__Mitsuokella
f__Lactobacillaceae.g__Lactobacillus
f__Pasteurellaceae
f__Pasteurellaceae.g__Actinobacillus
f__Alcaligenaceae
f__Fusobacteriaceae
f__Streptococcaceae.g__Streptococcus
f__Ruminococcaceae.g__Clostridium
f__Coriobacteriaceae.g__Eggerthella
f__Bacillaceae
f__Methanobacteriaceae.g__Methanobrevibacter
f__Peptostreptococcaceae.g__Clostridium
f__Clostridiaceae.g__Clostridium
f__Bifidobacteriaceae.g__Bifidobacterium
f__Oxalobacteraceae
f__Coriobacteriaceae.g__Olsenella
f__Lachnospiraceae.g__Roseburia
f__Paraprevotellaceae
Not_assigned
f__Ruminococcaceae
f__Lachnospiraceae
f__Ruminococcaceae.g__Oscillospira
f__Barnesiellaceae.g__Barnesiella
f__Rikenellaceae.g__Alistipes
f__Erysipelotrichaceae.g__Anaerorhabdus
f__Ruminococcaceae.g__Papillibacter
f__Lachnospiraceae.g__Marvinbryantia
f__Lachnospiraceae.g__Dorea
f__Campylobacteraceae.g__Campylobacter
f__Enterobacteriaceae
f__Bacteroidaceae.g__Bacteroides
f__Ruminococcaceae.g__Faecalibacterium
f__Eubacteriaceae.g__Pseudoramibacter_Eubacterium
f__Ruminococcaceae.g__Butyricicoccus
f__Coriobacteriaceae.g__Collinsella
f__Veillonellaceae
f__Veillonellaceae.g__Acidaminococcus
f__Paraprevotellaceae.g__Prevotella
f__Succinivibrionaceae.g__Succinivibrio
f__Prevotellaceae.g__Prevotella
f__Veillonellaceae.g__Succinispira
f__Erysipelotrichaceae
f__Desulfovibrionaceae.g__Desulfovibrio
f__Odoribacteraceae.g__Odoribacter
f__Ruminococcaceae.g__Subdoligranulum
f__Veillonellaceae.g__Selenomonas
3
2
1
0
-1
-2
-3
Successful
Unsuccessful

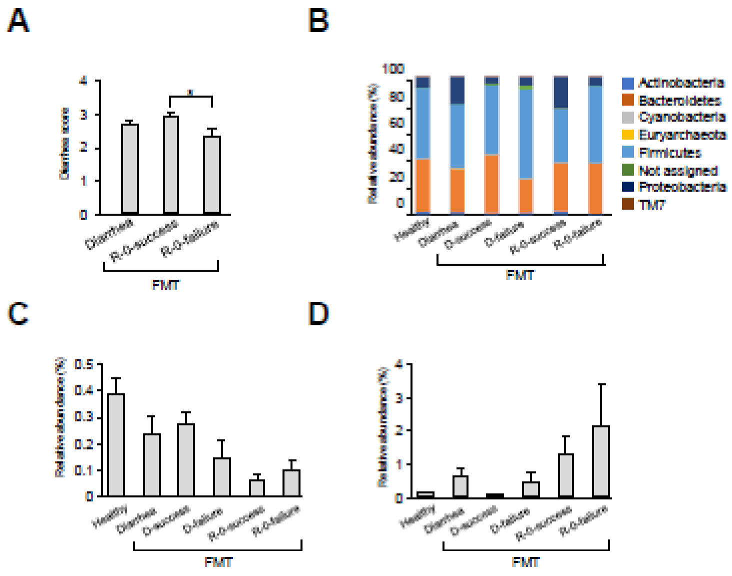
A
Diarrhea score
Diarrhea
R-0-success
R-0-failure
FMT
B
Relative abundance (%)
Healthy
Diarrhea
D-success
D-failure
R-0-success
R-0-failure
FMT
Actinobacteria
Bacteroidetes
Cyanobacteria
Euryarchaeota
Firmicutes
Not assigned
Proteobacteria
TM7
C
Relative abundance (%)
Healthy
Diarrhea
D-success
D-failure
R-0-success
R-0-failure
FMT
D
Relative abundance (%)
Healthy
Diarrhea
D-success
D-failure
R-0-success
R-0-failure
FMT
